# The Geography of Mental Health, Urbanicity, and Affluence

**DOI:** 10.3390/ijerph20085440

**Published:** 2023-04-07

**Authors:** Jeronimo Cortina, Shana Hardin

**Affiliations:** Department of Political Science and Population Health, University of Houston, Houston, TX 77204, USA; shardin4@cougarnet.uh.edu

**Keywords:** mental health, social groups, geography, geospatial statistics

## Abstract

Residential location has been shown to significantly impact mental health, with individuals in rural communities experiencing poorer mental health compared to those in urban areas. However, the influence of an individual’s social group on the relationship between residential location and mental health outcomes remains unclear. This study disaggregates the rural-urban binary and investigates how geography and social groupings interact to shape mental health outcomes. Merging data from PLACES and Claritas PRIZM, we conducted a hotspot analysis, generated bivariate choropleth maps, and applied multiscale geographically weighted regressions to examine the spatial distribution of mental health and social groupings. Our findings reveal that mental health is influenced by complex interactions, with social groups playing a critical role. Our study highlights that not all rural and urban areas are alike, and the extent to which social groups influence mental health outcomes varies within and across these areas. These results underscore the need for policies that are tailored to meet the unique mental health needs of individuals from different social groups in specific geographic locations to inform policy interventions that more effectively address mental health disparities across diverse communities.

## 1. Introduction

A recent article published in the *Journal of the American Medical Association* emphasizes the need for further research to comprehend the relationship between a person’s place of residence and their health outcomes [1]. While the call for research may appear obvious to some, the reality is quite complex. Where we reside, work, and spend our leisure time significantly influences the quantity and quality of opportunities available to us [2] as well as our behaviors [3]. Therefore, the question of whether geography impacts health outcomes lies at the heart of population health science, which aims to understand the root causes of health disparities across various populations to enhance health outcomes effectively [4]. These disparities vary significantly based on geography.

The existing literature suggests that an individual’s mental health outcomes are significantly influenced by the interplay between their characteristics and their place of residence. Factors such as socioeconomic status, physical environment, social support networks, and healthcare access have been identified as crucial determinants of mental health status [5,6,7,8,9,10,11,12]. These determinants are especially pronounced in urban versus rural settings. For instance, 59% of the decrease in community hospitals between 2015 and 2019 were rural hospitals [13], which limits the quantity and quality of opportunities for rural residents to focus on their health-related behaviors.

Prior research indicates that rural residents experience significantly worse mental health outcomes [14,15,16,17,18]. However, rural areas are not homogeneous, and their populations are complex and diverse. Although a significant portion of the U.S. population resides in rural areas, these individuals exhibit different demographic characteristics, social networks, and healthcare access. One study suggests that mental health disorders may be more prevalent in semi-rural areas than in rural areas [19], highlighting the importance of breaking down rural populations further to explain mental health outcomes, particularly as they relate to individuals’ social groups.

Using the latest release of the Population Level Analysis and Community Estimates (PLACES) (www.cdc.gov/places/ (accessed 20 February 2023)), a collaboration between the Centers for Disease Control and Prevention (CDC), the Robert Wood Johnson Foundation, and the CDC Foundation, we tackle this question by analyzing the spatial variation of the crude prevalence of mental health being not good for fourteen or more days among adults aged eighteen or more (hereinafter, mental health) using the Claritas PRIZM premier database, which classifies every U.S. household into fourteen social groupings (https://claritas.com/data/ (accessed 20 February 2023)) based on urbanicity and affluence.

This paper argues that not all rural and urban areas are created equal. It is here that using social group classifications provides a greater empirical and substantive understanding of how space shapes mental health outcomes. Existing studies contend that higher-income individuals tend to have improved mental health outcomes [7,8]; however, individuals within the most affluent social groups do not solely reside in urban areas. Those with higher incomes are dispersed throughout rural and urban settings; therefore, it is vital to understand the geographic distribution of social groupings to determine which rural areas have improved mental health outcomes while others continue to lag.

Rural and urban areas across the country vary by state and even within states. Houston, TX, for example, has an entirely different urban landscape than Dallas, TX. Alpine County, CA, with a population of 1235, and Prairie County, MT, with a population of 1091, both classified as two of the most rural counties in the country by the National Center for Health Statistics (NCHS) [20], cannot be more different. In Alpine County, CA, 43% of the population is part of a minority group, while in Prairie County, MT, only 17% is part of a minority group. The median value of owner-occupied housing units, 2017–2021, in Alpine, CA, is 3.2 times higher than in Prairie, MT [21].

Most researchers account for the differences between rural and urban places of residence. Such differences, most of the time, are based on population size. For example, the NCHS urban-rural classification divides U.S. counties into 6 tiers based on population size, from large metropolitan areas with a population of 1 million or more to noncore nonmetropolitan areas with less than 10,000 residents [20]. Indeed, the NCHS urban-rural classification helps identify differences in health outcomes across urbanization levels but perhaps is limited in identifying some of the social characteristics of the people that live in urban or rural areas.

This study aims to examine the relationship between geography, social groupings, and poor mental health days during the COVID-19 pandemic in 2020. The height of the pandemic offers a unique opportunity to study the impact of geography on mental health for two primary reasons. Firstly, while the pandemic affected everyone, its effects were not uniform, and geography can help us understand these differences. For instance, the pandemic led to significant mobility restrictions, resulting in reduced workplace, retail, recreation, and transit station mobility, as per Google’s mobility report, compared to pre-pandemic levels (https://www.google.com/covid19/mobility/ (accessed 20 February 2023)). However, the extent of these restrictions varied across locations and settings. Secondly, the pandemic brought unprecedented levels of uncertainty, as no one knew when it would end; how many lives it would claim; or the social, political, and economic costs it would incur. These factors also impacted various locations differently. Therefore, the combined impact of limited geographic mobility and pandemic-induced uncertainty provides a valuable context to comprehensively investigate the association between geography and mental health.

As adverse mental health outcomes persist, it is essential to prevent catch-all methods of addressing mental health crises that do not directly speak to specific population needs. Due to the unique composition of urban and rural areas, this study aims to showcase the geographic distribution of individuals throughout these areas who comprise divergent social groups. Without understanding who resides in these distinct areas, proper treatment and assessment of mental health problems cannot occur.

## 2. Materials and Methods

### 2.1. Data

We created a dataset by merging pre-existing data from PLACES and PRIZM. No Institutional Review Board (IRB) approval was required for the present study as the data used were pre-existing, de-identified, and publicly available. The final dataset contains 32,092 Zip Code Tabulation Areas (ZCTA). In total, we deleted 310 observations from the states of Alaska and Hawaii to better account for the neighboring spatial distribution of mental health and social groups. Keeping such states would produce unreliable results since such states do not border with those in the contiguous 48 adjoining U.S. states or those ZCTAs that lacked sufficient population to reliably estimate social groups [22,23].

#### 2.1.1. PLACES

The PLACES database provides model-based estimates of health measures. The model-based estimates were produced by the CDC using data sources such as the Behavioral Risk Factor Surveillance System (BRFSS) 2020, Census Bureau 2010 population estimates, and American Community Survey (ACS) 2015–2019 estimates. For this project, we focused on mental health as our dependent variable. The crude prevalence of the lack of health insurance, physical inactivity, and frequent physical health distress served as part of our explanatory variables (Table 1).

We included the explanatory variables listed in Table 1 as extant research determines they significantly impact an individual’s physical and mental health. First, lack of health insurance decreases access to mental health care, leading individuals with limited access to health insurance to worse mental health outcomes [24,25]. Next, current research has established a link between physical and mental health, showing that improvements in physical health and physical activity lead to improvements in mental health [26,27,28,29].

Although existing studies emphasize the importance of access to green and blue spaces for improving mental health [11,12,30], we excluded these variables from our analyses for two reasons. First, data on access to green and blue spaces are not readily available at the ZCTA level. The land cover data provided by the United States Environmental Protection Agency (EPA) specifically state the data should not be utilized for local-level analyses [31]. Second, due to the nature of the geographic data for separating different rural and urban areas based on social groupings, as a group becomes less urbanized, access to green and blue spaces is predicted to increase, leading to these variables being highly correlated. Therefore, we did not introduce a variable set to measure access to green or blue spaces, as this will result in multicollinearity.

#### 2.1.2. Claritas PRIZM Premier Social Groups

The 14 social groups of Claritas PRIZM Premier are constructed based on each place’s urbanicity class and affluence. First, each segment is placed in one of four urbanicity class categories (i.e., urban, suburban, second city, and town and rural, see Table 2 below). Second, within these urban classes, all the segments are classified based on affluence. Finally, all segments are grouped into one of the fourteen social groups. At the top of the affluence and density scales is group U1: Urban Uptown, in which residents live in urban areas and are very affluent. At the opposite extreme is group T4: Rustic living, where residents live in rural areas with a more downscale lifestyle. Table 2 summarizes the definitions for each of the 14 social groups, beginning with the highest affluence and density.

In addition, we extracted the average household expenditures on drugs, percent poverty status, and percent unemployed at the ZCTA level from Simply Analytics to serve as additional explanatory variables. First, since increases in drug prices can lead individuals to be unable to afford necessary medications to treat mental health issues, we included a measure of household expenditures on drugs [32,33,34,35]. Next, we utilized poverty status since those living in poverty experience worse mental health outcomes [36,37]. Lastly, unemployed individuals are more likely to struggle with mental health [38,39,40]. Table 3 summarizes the definitions.

### 2.2. Methods

The merged tabular cross-sectional database from PLACES and PIRZM was imported into Esri ArcGIS Pro version 3.1.0 to produce the spatial statistics described below to learn about the spatial distribution of mental health given our control variables in each of the fourteen social groups, which is akin to fitting an interaction model between the controls and the social groups, with the advantage that the interpretation of the results is more straightforward [41].

Although there is no single cause for mental health distress, the explanatory variables included in this paper attempt to measure the most common associated predictors [24,25,26,27,28,29,32,33,34,35,36,37,38,39,40,42,43]. Since our level of analysis is at the ZCTA level, we solely focused our study on the spatial distribution and impact of such variables on mental health’s geographic patterns and made no inferences at the individual level to avoid ecological fallacy issues.

First, we estimated the Getis-Ord Gi* statistic to analyze if frequent mental health distress clusters spatially via the identification of hotspots. A hotspot compares an area (i.e., ZCTA) with a high concentration of poor mental health days with the expected number given a random distribution. The Gi* statistic compares the density within a ZCTA with a random spatial model and measures the interaction with other areas to understand the occurrence of spatial patterns [44]. In other words, it analyzes if the crude prevalence of frequent mental health distress in a particular ZCTA is high or low, given the crude prevalence of neighboring ZCTAs. A statistically significant hot spot is one in which there is a high value of frequent mental health distress in a particular zip code surrounded by other zip codes with high values (i.e., larger z-scores); in contrast, a cold spot would have smaller z-scores suggesting a significant clustering of a low prevalence of frequent mental health distress.

Second, we presented a series of bivariate choropleth maps to show the spatial distribution between the crude prevalence of frequent mental health distress and social groups. The bivariate coding scheme represents the product of each variable with three discrete classes (i.e., low, medium, and high) to create a grid of nine unique colors [44]. Using Stata 17, we performed a Kruskal-Wallist test to determine if the median crude prevalence of frequent mental health distress was the same across social groups.

Finally, we estimated a series of multiscale geographically weighted regressions (MGWR) by social group to model the spatial correlations between mental health and the macro social determinants of health to determine which of our explanatory variables has the most robust association with mental health while controlling for the other predictors. MGWR is a derivation of geographically weighted regression (GWR) that constructs a linear regression between a dependent variable and a set of explanatory variables within a neighborhood. The main difference between GWR and MGWR is that in the latter, the scale of analysis can vary between variables in contrast with the former, which assumes that each explanatory variable’s scale is identical [45].

## 3. Results

### 3.1. Hotspot Analysis

**Figure 1:**
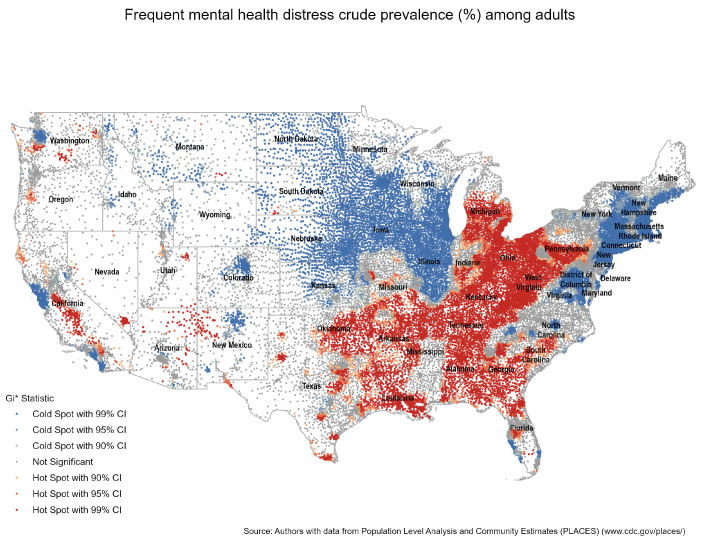
Hot Spot Analysis for Mental Health.

Figure 1 shows significant clustering of the crude prevalence of frequent mental health distress in the South (e.g., parts of Louisiana, Mississippi, Alabama, Georgia, and South Carolina), Coal Country (e.g., parts of Tennessee, West Virginia, and Pennsylvania), and the Rust Belt (e.g., parts of Ohio, Pennsylvania, Michigan, and Indiana). In addition, certain parts of Oklahoma, Arkansas, and East Texas; cities such as Dallas-Fort Worth and Houston; and California’s San Joaquin Valley also experience significant spatial clustering—indicated by the red dots. In contrast, in the Great Plains (e.g., the Dakotas, Minnesota, and Iowa), around the Great Lakes (e.g., Michigan and Wisconsin), the North East, northern New Mexico, Colorado, and parts of Washington and California, among other areas in the country, the prevalence of frequent mental health distress seems to be lower—indicated by the blue dots that illustrate ZCTAs where there is no high crude prevalence. In the rest of the country, there is no significant geographic concentration of the crude prevalence of frequent mental health distress—as seen with the gray dots (All Figures are available in the Supplementary Materials).

To account for the Modifiable Areal Unit Problem (MAUP), which concerns the impact of spatial unit selection on the statistical analysis of spatial data, we conducted a comparison of the spatial distribution of mental health using county and ZCTA-level data. Specifically, we calculated Moran’s I Statistic and performed a hotspot analysis. The results of both analyses indicated a statistically significant clustered pattern, demonstrating that despite different levels of aggregation, mental health exhibits similar spatial patterns between counties and ZCTAs (See File S1 for complete results).

### 3.2. Bivariate Choropleth Maps

**Figure 2:**
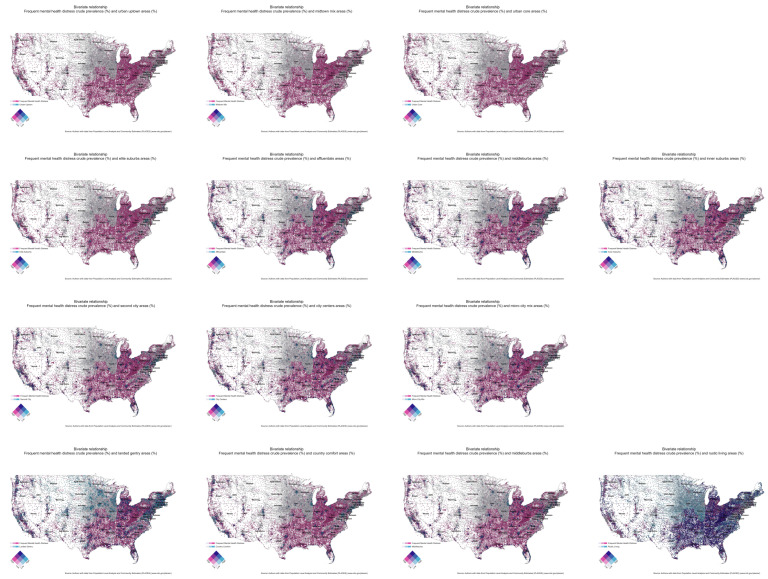
Bivariate choropleth maps between the crude prevalence of frequent mental health distress and individual social groups. A pink shade indicates a high spatial concentration of frequent mental health distress, while a light blue indicates a high geographic presence of a particular social group. A dark blue shade suggests a high spatial concentration of both frequent mental health and a particular social group.

The bivariate choropleth maps (Figure 2) reveal various geospatial relationships. We observed negative and small correlations between frequent mental health distress and the distribution of the most urban and/or affluent ZCTAs (p_MH-U1_ = −0.11, p_MH-S1_ = −0.26, p_MH-C1_ = −0.05, p_MH-T1_ = −0.30). In contrast, the correlation between mental health distress and the distribution of less urban and less affluent ZCTAs is mixed. Specifically, we find positive and somewhat stronger correlations in C2, C3, and T4 social groups (p_MH-C2_ = 0.19, p_MH-C3_ = 0.20, p_MH-T4_ = 0.39). Despite the apparent spatial consistency of frequent mental health distress across social groups, the Kruskal-Wallist test revealed that the median crude prevalence of frequent mental health distress was not uniform across the fourteen social groupings (χ2 = 10,866.37, *p* = 0.0001).

### 3.3. Multiscale Geographically Weighted Regressions

Table 4 shows the scaled mean value of each coefficient by social grouping with its standard deviation in parenthesis (results available in File S1). The mean value reflects the association between each explanatory variable and the crude prevalence of mental health distress. Positive mean values indicate that an increase in the explanatory variable is associated with an increase in the crude prevalence of frequent mental health distress. In contrast, negative mean values indicate that a rise in one of the explanatory variables is related to a decrease in the frequency of mental health distress. The standard deviation indicates each explanatory variable’s spatial variation. For example, a low standard deviation suggests low spatial variability and vice versa.

In most social groups (except U2, C1, C2, and C3), the lack of health insurance among adults aged 18–64 years has the strongest association with frequent mental health distress. Regular physical health distress is the second explanatory variable with a powerful effect on mental health in most social groups (with exceptions in U2, S3, S4, C2, and C3). Physical inactivity is negatively related to mental health in all social groups, except for S3, S4, and C3, where the relationship is positive.

When comparing coefficients by urbanicity and affluence, the results are mixed. In the most affluent and urban areas (U1 and S1), health insurance, frequent physical distress, and unemployment have the highest mean coefficients. However, in urban but less affluent ZCTAs (U3 and S4), the lack of health insurance remains the explanatory variable with the strongest relationship with mental health distress. For U3, frequent mental health distress is closely followed by poverty, while poverty is the second explanatory variable for inner suburbs ZCTAs (S4). Lack of physical activity has the third most substantial impact on mental health for S4.

In the most rural and less affluent ZCTAs (T3 and T4), lack of health insurance, frequent physical distress, and poverty have the highest mean values, following similar patterns as urban core ZCTAs (U3). Among the explanatory variables, lack of health insurance has the highest spatial variation across our study area, followed by physical inactivity, frequent physical health distress, average household drug expenditures, poverty, and unemployment. In U1, physical inactivity has the highest spatial variation, while in T1, lack of health insurance displays the most extensive spatial variation. Lack of health insurance has the highest spatial variation in T4, while U3 has the lowest.

From the results presented in Table 4, we can conclude that the lack of health insurance is the variable with the most robust relationship with frequent mental health distress at the ZCTA level. To further explore their spatial relationship, we investigated the variation in the local parameter estimates of the lack of health insurance for each social group’s mental health distress.

**Figure 3:**
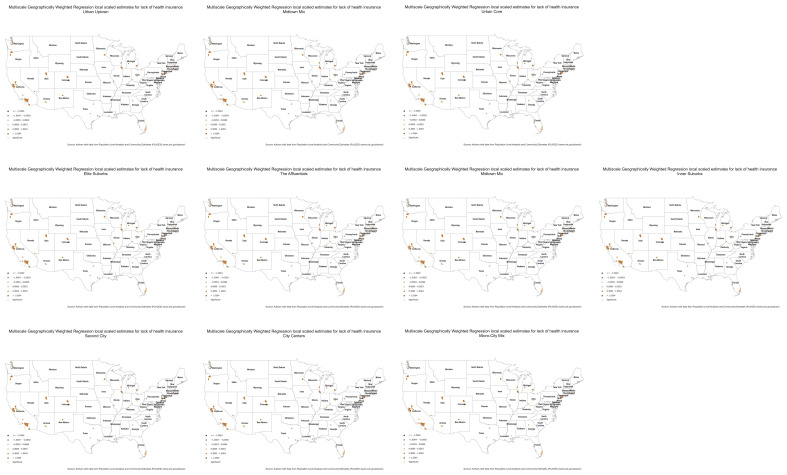

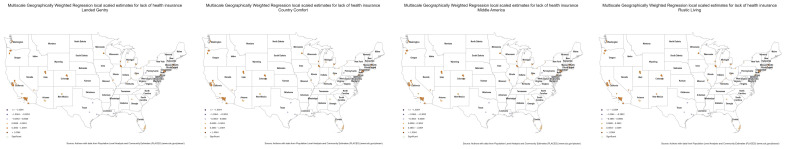
Multiscale Geographically Weighted Regression local scaled estimates for lack of healthcare by social group. Brown dots indicate a highly positive impact of the lack of health insurance on mental health, while purple dots indicate a negative relationship.

Figure 3 displays the coefficient estimate with a divergent color scheme centered at zero to identify where the lack of health insurance has a positive or negative relationship with frequent mental health distress. The green halos indicate statistically significant associations with 95 percent confidence.

Overall, we can observe significant spatial variation within and between social groups, most notably in less urban areas. For example, the lack of health insurance in T2 ZCTAs exhibit a positive relationship (i.e., brown dots) in some locations and a negative relationship in others (i.e., purple dots). In terms of statistical significance, there is also crucial spatial variation; however, in rural areas, the impact of the lack of health insurance on frequent mental health distress seems to be the strongest, as well as in the upper rungs of suburban areas.

## 4. Discussion

We note four important findings in our study. First, not all rural areas and not all urban areas are alike. The bivariate maps illustrated a discordance in geographic distributions between social groups and mental health. In the most rural and less affluent areas, the correlation between individuals’ residence and the prevalence of mental health distress is strong. However, it is also here where we recognize that some suburban areas also experience significant mental health distress, not solely the most remote and least affluent areas; therefore, future studies need to explore why suburban areas, even with increased mental health resources, still experience poorer mental health outcomes.

Second, our study finds that the strength of the impact of each explanatory variable on the crude prevalence of frequent mental health distress behaves differently in each social group. As the previous literature has found [24,25], the lack of health insurance is positively related to an increase in frequent mental health distress; however, its impact is more substantial in less urban and less affluent areas (i.e., T3 and T4), followed by rural and affluent areas (i.e., T1 and T2), urban and affluent ZCTAs (i.e., U1, S1, and C1), and the urban and less affluent ZCTAs (i.e., U3, S4, and C3). In addition, the impact of other factors such as physical health, unemployment, poverty, and physical inactivity changes significantly depending on the social group and location.

At first glance, the negative relationship between physical inactivity and mental health may seem counterintuitive [46,47,48,49]; however, the impact of physical activity on mental health cannot be understood in a vacuum but as another explanatory variable in the context of the general model during a global pandemic, in which, everyday routines were significantly altered. In addition, the effects of physical activity on mental health may be specific to the characteristics of physical activity, such as intensity, duration, and type of exercise, rather than just physiological activation [50] as well as the location where these activities occur. PLACES aggregates data from individual survey questions, which may lead to effects on the wording of the questions. In this case, the question used to estimate the lack of physical inactivity was based on a range of activities that may have different effects on mental health, namely running, calisthenics, golf, gardening, or walking. Finally, researchers must be careful not to commit the atomistic fallacy that occurs when incorrect inferences are made about relationships in aggregate data based on relationships observed in individual data.

Third, our geospatial results suggest that although levels of frequent mental health distress are commonly found in the most remote areas, mental health distress is also found in other communities with greater affluence; therefore, rural communities should not be considered one group since the levels of mental health distress diverge. This finding underscores the need to increase our understanding of geography and social determinants of health. In addition, as existing studies contend that mental health outcomes are worse in rural areas [14,15,16,17,18,19], these studies do not account for the fact that not all affluent individuals are concentrated in urban areas. These individuals also reside in rural areas and have better access to mental health services than those who live in less affluent areas; therefore, it is necessary to break up rural areas to assess mental health treatments and services.

Finally, the divergent spatial patterns between frequent mental health distress and the explanatory variables used in this study highlight a need for policy to tailor better mental health prevention that emphasizes meeting the needs of target populations in specific geographies.

While our study provides valuable insights, there are several limitations that should be acknowledged. First, the dynamic nature of the COVID-19 pandemic means that mental health outcomes are subject to change over time as new interventions are implemented. Therefore, our findings should not be generalizable to the entire duration of the pandemic. Second, the mapping analyses relied on some model-based estimates, which inherently contain some uncertainty. As such, our findings cannot be interpreted as causal or determinant. Lastly, our analysis utilized PLACES data collected at different time points, which may introduce temporal bias into our results.

Despite these limitations, our study offers a “big picture” framework that may help to guide future population and mental health services planning. The maps presented in this study provide important geospatial input data for public policy planning, especially as they relate to non-health factors—such as social activities, spending habits, and lifestyle preferences—that can influence health outcomes.

Overall, the results from this study provide implications for future research and how to address mental health crises. First, future research should use more comprehensive coding schemes when analyzing rural and urban populations. Additionally, evaluations should include affluence as wealth, which can lead to better healthcare access. Next, given these results, existing catch-all methods utilized to address mental health problems should be expanded to include more appropriate measures for rural and urban communities. Diverse populations need diverse treatments, and existing studies are missing part of the picture.

## 5. Conclusions

Extant research has improved our understanding of geographic location’s role in mental health outcomes. Results from this study conclude that urban and rural areas are complex and diverse, leading to different mental health outcomes for individuals within these areas that are dependent upon one’s social group. Given these findings, policies attempting to alleviate mental health issues within urban and rural communities should further separate these groups and consider how other factors account for persistent mental health problems.

In sum, these findings highlight a need for breaking up large, classified groups before determining ways to address mental health and insurance access on a larger scale. Future studies should continue to utilize a multifaceted urban and rural coding scheme to construct policies targeting these complex groups and their mental health needs.

## Figures and Tables

**Table 1:** PLACES Variable Definitions.

Variable	Definition
Mental Health Not Good For ≥14 Days Among Adults Aged ≥18 Years	Respondents aged ≥18 years who reported 14 or more days during the past 30 days during which their mental health was not good.
Annual Prevalence of Current Lack Of Health Insurance Among Adults Aged 18–64 Years	Respondents aged 18–64 years who report having no current health insurance coverage
Annual Prevalence of No Leisure-Time Physical Activity Among Adults Aged ≥18 Years	During the past month, other than your regular job, did you participate in any physical activities or exercises such as running, calisthenics, golf, gardening, or walking for exercise?
Physical Health Not Good For ≥14 Days Among Adults Aged ≥18 Years	Respondents aged ≥18 years who reported 14 or more days during the past 30 days during which their physical health was not good.

**Table 2:** Claritas PRIZM Premier Social Group Definitions.

Social Group	Definition
Urban Uptown (U1)	Home to the most affluent individuals living in the city core, who are able to purchase luxury goods and vacation abroad regularly
Midtown Mix (U2)	Home to those who are younger and have active social lives within the city, leading them to spend money frequently at bars and restaurants while also having the ability to purchase new consumer electronics
Urban Core (U3)	Contains individuals with more modest incomes; therefore, they are more likely to live in apartments within the city and have less disposable income for eating out and purchasing goods
Elite Suburbs (S1)	Where individuals with six-figure incomes and large homes reside; these individuals spend their money on expensive clothes, cars, and vacations
The Affluentials (S2)	Enjoy comfortable living in the suburbs and have white-collar jobs; they consistently buy healthier foods and computer equipment
Middleburbs (S3)	Consist of individuals who are homeowners in the suburbs that shop at midscale department stores and regularly eat at casual-dining restaurants
Inner Suburbs (S4)	Home to a mix of young and retired individuals who can be homeowners or renters; these individuals have downscale lifestyles and do not have the ability to eat out or shop regularly
Second City Society (C1)	Comprises individuals who live outside of the urban core, with large homes and holding executive jobs; residents also spend more on casual dining and upscale retailers
City Centers (C2)	Home to those in satellite cities who are middle class and regularly go to movie theaters and bowling alleys
Micro-City Mix (C3)	Consists of downscale, blue-collar residents who do not have readily available disposable income for dining, activities, and goods
Landed Gentry (T1)	Residents live in smaller towns but have large homes, college degrees, and professional jobs; these individuals spend their disposable incomes on cars and recreational equipment (e.g., powerboats and four-wheelers)
Country Comfort (T2)	Home to upper-middle-class individuals who regularly participate in outdoor activities, woodworking, and crafting and prefer to own larger trucks and SUVs
Middle America (T3)	Residents are middle to lower-class individuals who prefer fishing, hunting, and meeting at civic clubs; in these remote areas, high school football is a main source of entertainment
Rustic Living (T4)	Residents live in the most remote towns and have modest incomes; these individuals spend their leisure time participating in small-town activities, such as social groups at local churches, veterans’ clubs, and car racing

**Table 3:** Simply Analytics Variable Definitions.

Variable	Definition
Household Average Expenditures on Drugs	The household average cost of drugs, which includes prescription and nonprescription drugs
Percent Poverty Status	The percentage of the population living in poverty
Percent Unemployed Civilian Labor Force	The percentage of the population that is unemployed

**Table 4:** Mean and standard deviations of coefficient estimates across social groupings.

	Intercept	Total Pop.	Lack of Health Insurance	Physical Inactivity	Physical Health Not Good	Household Avg. Expenditures on Drugs	% Poverty Status	% Unemployed Civilian Labor Force
Urban Uptown (U1)	−0.06 (0.38)	0.03 (0.09)	0.55 (0.33)	−0.74 (0.47)	0.44 (0.29)	−0.52 (0.26)	−0.17 (0.21)	0.07 (0.12)
Midtown Mix (U2)	0.003 (0.20)	0.004 (0.09)	0.33 (0.40)	−0.44 (0.31)	0.28 (0.28)	−0.37 (0.30)	0.38 (0.15)	0.13 (0.19)
Urban Core (U3)	0.13 (0.48)	0.09 (0.12)	0.41 (0.56)	−0.29 (0.85)	0.36 (0.71)	−0.27 (0.23)	0.31 (0.19)	0.11 (0.15)
Elite Suburbs (S1)	0.32 (0.65)	0.004 (0.08)	1.25 (0.67)	−0.78 (0.41)	0.36 (0.20)	−0.40 (0.17)	0.06 (0.16)	0.10 (0.13)
The Affluentials (S2)	−0.09 (0.49)	0.04 (0.10)	0.61 (0.47)	−0.18 (0.27)	0.20 (0.22)	−0.38 (0.23)	0.10 (0.16)	0.03 (0.17)
Middleburbs (S3)	0.15 (0.33)	−0.01 (0.12)	0.27 (0.26)	0.06 (0.39)	0.03 (0.39)	−0.51 (0.17)	0.13 (0.13)	0.06 (0.11)
Inner Suburbs (S4)	0.23 (0.33)	0.002 (0.17)	0.49 (0.45)	0.17 (0.46)	−0.37 (0.60)	−0.13 (0.45)	0.27 (0.40)	0.06 (0.19)
Second City (C1)	0.05 (0.10)	−0.01 (−0.01)	0.29 (0.11)	−0.58 (0.13)	0.93 (0.23)	−0.85 (0.19)	−0.07 (0.04)	0.02 (0.19)
City Centers (C2)	0.08 (0.39)	0.04 (0.10)	0.20 (0.37)	−0.06 (0.43)	0.10 (0.37)	−0.62 (0.26)	0.34 (0.16)	0.04 (0.12)
Micro-City Mix (C3)	0.08 (0.43)	−0.05 (0.16)	0.16 (0.43)	0.21 (0.67)	0.08 (0.52)	−0.32 (0.30)	0.20 (0.24)	0.05 (0.12)
Landed Gentry (T1)	0.18 (0.59)	0.004 (0.11)	0.82 (0.64)	−0.01 (0.34)	0.12 (0.28)	−0.21 (0.23)	0.05 (0.13)	0.03 (0.10)
Country Comfort (T2)	0.22 (0.81)	0.06 (0.19)	1.03 (0.64)	−0.08 (.45)	0.06 (0.31)	−0.02 (0.13)	0.02 (0.09)	0.01 (0.09)
Middle America (T3)	0.28 (0.93)	0.05 (0.13)	1.11 (0.74)	−0.23 (0.56)	0.19 (0.40)	−0.02 (0.13)	0.04 (0.09)	0.04 (0.10)
Rustic Living (T4)	0.21 (1.06)	0.04 (0.13)	0.84 (0.66)	−0.12 (0.51)	0.23 (0.42)	−0.05 (0.16)	0.06 (0.11)	0.03 (0.11)

## Data Availability

Two main data sources were used in this study. Publicly available datasets used in this study can be found here: https://chronicdata.cdc.gov/500-Cities-Places/PLACES-ZCTA-Data-GIS-Friendly-Format-2022-release/kee5-23sr (accessed 20 February 2023). Restrictions apply to the following. Data was obtained from Simply Analytics and are available from the author at jcortina@cougarnet.uh.edu with the permission of Simply Analytics.

## Supplementary Materials

The following supporting information can be downloaded at: https://www.mdpi.com/article/10.3390/ijerph20085440/s1, Figure S1: Hot Spot Analysis for Mental Health; Figure S2: Bivariate Choropleth Maps; Figure S3: Multiscale Geographically Weighted Regressions. File S1: MGWR output and Moran’s I Statistic sensitivity analysis.
