# Supplementary material for: The Geography of Mental Health, Urbanicity, and Affluence
Source: Int J Environ Res Public Health. 2023 Apr 7;20(8):5440. doi: 10.3390/ijerph20085440 (PMC10138034; doi:10.3390/ijerph20085440)

Social Group	Definition
Urban Uptown (U1)	Home to the most affluent individuals living in the city core, who are able to purchase luxury goods and vacation abroad regularly
Midtown Mix (U2)	Home to those who are younger and have active social lives within the city, leading them to spend money frequently at bars and restaurants while also having the ability to purchase new consumer electronics
Urban Core (U3)	Contains individuals with more modest incomes; therefore, they are more likely to live in apartments within the city and have less disposable income for eating out and purchasing goods
Elite Suburbs (S1)	Where individuals with six-figure incomes and large homes reside; these individuals spend their money on expensive clothes, cars, and vacations
The Affluentials (S2)	Enjoy comfortable living in the suburbs and have white-collar jobs; they consistently buy healthier foods and computer equipment
Middleburbs (S3)	Consist of individuals who are homeowners in the suburbs that shop at midscale department stores and regularly eat at casual-dining restaurants
Inner Suburbs (S4)	Home to a mix of young and retired individuals who can be homeowners or renters; these individuals have downscale lifestyles and do not have the ability to eat out or shop regularly
Second City Society (C1)	Comprised of individuals who live outside of the urban core, with large homes and holding executive jobs; residents also spend more on casual dining and upscale retailers
City Centers (C2)	Home to those in satellite cities who are middle class and regularly go to movie theaters and bowling alleys
Micro-City Mix (C3)	Consists of downscale, blue-collar residents who do not have readily available disposable income for dining, activities, and goods
Landed Gentry (T1)	Residents live in smaller towns but have large homes, college degrees, and professional jobs; these individuals spend their disposable incomes on cars and recreational equipment (e.g., powerboats and four-wheelers)
Country Comfort (T2)	Home to upper-middle-class individuals who regularly participate in outdoor activities, woodworking, and crafting and prefer to own larger trucks and SUVs
Middle America (T3)	Residents are middle to lower-class individuals who prefer fishing, hunting, and meeting at civic clubs; in these remote areas, high school football is a main source of entertainment
Rustic Living (T4)	Residents live in the most remote towns and have modest incomes; these individuals spend their leisure time participating in small-town activities, such as social groups at local churches, veterans' clubs, and car racing

Variables	
TOTALPOPUL	Total Population 2020
LACK_HEALT	Lack of health insurance
PHYSICAL_I	Physical inactivity
FREQUENT_P	Frequent physical health distress
DRUGS	Household Avg. expenditure on drugs
PVTY_AGE	Percent poverty
UNEMP	Percent unemployed of the civilian labor force

U1: Summary Statistics for Coefficients Estimates

Explanatory Variables	Mean	Standard Deviation	Minimum	Median	Maximum
Intercept	-0.06	0.38	-0.9354	-0.1017	0.914
TOTALPOPUL	0.03	0.09	-0.2124	0.0279	0.3209
LACK_HEALT	0.55	0.33	-0.2827	0.6281	1.2249
PHYSICAL_I	-0.74	0.47	-1.5358	-0.5458	-0.0341
FREQUENT_P	0.44	0.29	-0.2528	0.5005	0.8671
DRUGS	-0.52	0.26	-1.0286	-0.5495	0.0631
PVTY_AGE	0.17	0.21	-0.365	0.2222	0.5908
UNEMP	0.07	0.12	-0.3468	0.05	0.3894

Model Diagnostics

Statistic	MGWR
R-Squared	0.8848
Adjusted R-Squared	0.8356
AICc	809.0027
Sigma-Squared	0.1643
Sigma-Squared MLE	0.1152
Effective Degrees of Freedom	369.0298

Summary of Explanatory Variables and Neighborhoods

Explanatory Variables	Neighbors (% of Features)[a]	Significant (% of Features)[b]
Intercept	31 (5.89)	102 (19.39)
TOTALPOPUL	50 (9.51)	0 (0.00)
LACK_HEALT	50 (9.51)	351 (66.73)
PHYSICAL_I	50 (9.51)	415 (78.90)
FREQUENT_P	50 (9.51)	382 (72.62)
DRUGS	50 (9.51)	378 (71.86)
PVTY_AGE	50 (9.51)	157 (29.85)
UNEMP	50 (9.51)	63 (11.98)

a: This number in the parenthesis ranges from 0 to 100%, and can be interpreted as a local, regional, global scale based on the geographical context from low to high.

b: In the parentheses, the percentage of features that have significant coefficients of an explanatory variable.

Optimal Bandwidths Search History

Iterations	Intercept	TOTALPOPUL	LACK_HEALT	PHYSICAL_I	FREQUENT_P	DRUGS	PVTY_AGE	UNEMP	AICc
1	30	50	50	50	50	50	50	50	883.6
2	30	50	50	50	50	50	50	50	851
3	30	50	50	50	50	50	50	50	837.4
4	30	50	50	50	50	50	50	50	830.1
5	30	50	50	50	50	50	50	50	825.4
6	30	50	50	50	50	50	50	50	822.1
7	30	50	50	50	50	50	50	50	819.7
8	30	50	50	50	50	50	50	50	817.7
9	31	50	50	50	50	50	50	50	814.7
10	31	50	50	50	50	50	50	50	813.1
11	31	50	50	50	50	50	50	50	811.8
12	31	50	50	50	50	50	50	50	810.6
13	31	50	50	50	50	50	50	50	809.7
14	31	50	50	50	50	50	50	50	809

Bandwidth Statistics Summary

Explanatory Variables	Optimal Number of Neighbors	Effective Number of Parameters	Adjusted Value of Alpha	Adjusted Critical Value of Pseudo-t Statistics
Intercept	31	32.2	0.0016	3.1884
TOTALPOPUL	50	20.1	0.0025	3.0458
LACK_HEALT	50	15.4	0.0032	2.9627
PHYSICAL_I	50	12.95	0.0039	2.9078
FREQUENT_P	50	16.09	0.0031	2.9765
DRUGS	50	17.55	0.0028	3.0037
PVTY_AGE	50	20.53	0.0024	3.0523
UNEMP	50	22.15	0.0023	3.0756

U2: Summary Statistics for Coefficients Estimates

Explanatory Variables	Mean	Standard Deviation	Minimum	Median	Maximum
Intercept	0.00	0.20	-0.4408	0.0199	0.4585
TOTALPOPUL	0.00	0.09	-0.2071	-0.0091	0.2422
LACK_HEALT	0.33	0.40	-0.6225	0.3414	0.9833
PHYSICAL_I	-0.44	0.31	-1.0865	-0.388	0.2282
FREQUENT_P	0.28	0.28	-0.1202	0.2342	0.8784
DRUGS	-0.37	0.30	-1.013	-0.2588	0.0742
PVTY_AGE	0.38	0.15	0.0391	0.3728	0.7058
UNEMP	0.13	0.19	-0.384	0.1389	0.6281

Model Diagnostics

Statistic	MGWR
R-Squared	0.8739
Adjusted R-Squared	0.8218
AICc	721.4655
Sigma-Squared	0.178
Sigma-Squared MLE	0.1261
Effective Degrees of Freedom	318.599

Summary of Explanatory Variables and Neighborhoods

Explanatory Variables	Neighbors (% of Features)[a]	Significant (% of Features)[b]
Intercept	35 (7.78)	1 (0.22)
TOTALPOPUL	50 (11.11)	0 (0.00)
LACK_HEALT	50 (11.11)	192 (42.67)
PHYSICAL_I	47 (10.44)	309 (68.67)
FREQUENT_P	49 (10.89)	167 (37.11)
DRUGS	50 (11.11)	113 (25.11)
PVTY_AGE	49 (10.89)	245 (54.44)
UNEMP	50 (11.11)	27 (6.00)

a: This number in the parenthesis ranges from 0 to 100%, and can be interpreted as a local, regional, global scale based on the geographical context from low to high.

b: In the parentheses, the percentage of features that have significant coefficients of an explanatory variable.

Optimal Bandwidths Search History

Iterations	Intercept	TOTALPOPUL	LACK_HEALT	PHYSICAL_I	FREQUENT_P	DRUGS	PVTY_AGE	UNEMP	AICc
1	30	50	50	50	50	50	50	50	797.5
2	30	50	50	50	50	50	49	50	770.5
3	35	50	50	50	49	50	49	50	757.8
4	35	50	50	47	49	50	49	50	748.2
5	35	50	50	47	49	50	49	50	740.8
6	35	50	50	47	49	50	49	50	735.2
7	35	50	50	47	49	50	49	50	730.8
8	35	50	50	47	49	50	49	50	727.5
9	35	50	50	47	49	50	49	50	725
10	35	50	50	47	49	50	49	50	723.3
11	35	50	50	47	49	50	49	50	722.2
12	35	50	50	47	49	50	49	50	721.5

Bandwidth Statistics Summary

Explanatory Variables	Optimal Number of Neighbors	Effective Number of Parameters	Adjusted Value of Alpha	Adjusted Critical Value of Pseudo-t Statistics
Intercept	35	24.26	0.0021	3.1069
TOTALPOPUL	50	18.01	0.0028	3.015
LACK_HEALT	50	14.1	0.0035	2.9378
PHYSICAL_I	47	13.16	0.0038	2.9158
FREQUENT_P	49	12.14	0.0041	2.8897
DRUGS	50	14.42	0.0035	2.945
PVTY_AGE	49	17.04	0.0029	2.9977
UNEMP	50	18.28	0.0027	3.0196

U3: Summary Statistics for Coefficients Estimates

Explanatory Variables	Mean	Standard Deviation	Minimum	Median	Maximum
Intercept	0.13	0.48	-0.8214	0.0241	1.6166
TOTALPOPUL	0.09	0.12	-0.1385	0.0711	0.4336
LACK_HEALT	0.41	0.56	-1.3402	0.5155	1.546
PHYSICAL_I	-0.29	0.85	-1.991	-0.4106	1.3232
FREQUENT_P	0.36	0.71	-1.4086	0.5038	1.3025
DRUGS	-0.27	0.23	-0.628	-0.3162	0.2551
PVTY_AGE	0.31	0.19	-0.1852	0.3364	0.8338
UNEMP	0.11	0.15	-0.1202	0.0585	0.8224

Model Diagnostics

Statistic	GWR	MGWR
R-Squared	0.9314	0.9374
Adjusted R-Squared	0.8912	0.9058
AICc	1084.1247	1054.1923
Sigma-Squared	0.1087	0.0942
Sigma-Squared MLE	0.0686	0.0626
Effective Degrees of Freedom	613.8873	646.3472

Optimal GWR Bandwidth: 50 (K nearest neighbors).

Summary of Explanatory Variables and Neighborhoods

Explanatory Variables	Neighbors (% of Features)[a]	Significant (% of Features)[b]
Intercept	30 (3.08)	128 (13.16)
TOTALPOPUL	50 (5.14)	73 (7.50)
LACK_HEALT	30 (3.08)	254 (26.10)
PHYSICAL_I	30 (3.08)	393 (40.39)
FREQUENT_P	30 (3.08)	425 (43.68)
DRUGS	50 (5.14)	374 (38.44)
PVTY_AGE	50 (5.14)	296 (30.42)
UNEMP	50 (5.14)	54 (5.55)

a: This number in the parenthesis ranges from 0 to 100%, and can be interpreted as a local, regional, global scale based on the geographical context from low to high.

b: In the parentheses, the percentage of features that have significant coefficients of an explanatory variable.

Optimal Bandwidths Search History

Iterations	Intercept	TOTALPOPUL	LACK_HEALT	PHYSICAL_I	FREQUENT_P	DRUGS	PVTY_AGE	UNEMP	AICc
0	50	50	50	50	50	50	50	50	1084
1	30	50	30	30	30	50	50	50	1107
2	30	50	30	30	30	50	50	50	1070
3	30	50	30	30	30	50	50	50	1059
4	30	50	30	30	30	50	50	50	1054

Bandwidth Statistics Summary

Explanatory Variables	Optimal Number of Neighbors	Effective Number of Parameters	Adjusted Value of Alpha	Adjusted Critical Value of Pseudo-t Statistics
Intercept	30	48.19	0.001	3.2951
TOTALPOPUL	50	37.14	0.0013	3.22
LACK_HEALT	30	47.49	0.0011	3.291
PHYSICAL_I	30	47.68	0.001	3.2921
FREQUENT_P	30	43.79	0.0011	3.2676
DRUGS	50	34.14	0.0015	3.1954
PVTY_AGE	50	32.08	0.0016	3.1771
UNEMP	50	36.14	0.0014	3.212

S1: Summary Statistics for Coefficients Estimates

Explanatory Variables	Mean	Standard Deviation	Minimum	Median	Maximum
Intercept	0.32	0.65	-0.8855	0.3463	2.1132
TOTALPOPUL	0.00	0.08	-0.2481	0.0047	0.2133
LACK_HEALT	1.25	0.67	0.0305	1.1912	2.8495
PHYSICAL_I	-0.78	0.41	-1.6826	-0.7969	0.1717
FREQUENT_P	0.36	0.20	-0.3037	0.404	0.6936
DRUGS	-0.40	0.17	-0.908	-0.3733	-0.0597
PVTY_AGE	0.06	0.16	-0.3337	0.0354	0.5413
UNEMP	0.10	0.13	-0.1526	0.069	0.547

Model Diagnostics

Statistic	GWR	MGWR
R-Squared	0.8803	0.8889
Adjusted R-Squared	0.8081	0.8366
AICc	827.5506	791.8382
Sigma-Squared	0.1917	0.1632
Sigma-Squared MLE	0.1197	0.1111
Effective Degrees of Freedom	310.9796	339.0367

Optimal GWR Bandwidth: 50 (K nearest neighbors).

Summary of Explanatory Variables and Neighborhoods

Explanatory Variables	Neighbors (% of Features)[a]	Significant (% of Features)[b]
Intercept	30 (6.02)	185 (37.15)
TOTALPOPUL	50 (10.04)	0 (0.00)
LACK_HEALT	38 (7.63)	319 (64.06)
PHYSICAL_I	48 (9.64)	312 (62.65)
FREQUENT_P	50 (10.04)	191 (38.35)
DRUGS	50 (10.04)	233 (46.79)
PVTY_AGE	50 (10.04)	35 (7.03)
UNEMP	50 (10.04)	26 (5.22)

a: This number in the parenthesis ranges from 0 to 100%, and can be interpreted as a local, regional, global scale based on the geographical context from low to high.

b: In the parentheses, the percentage of features that have significant coefficients of an explanatory variable.

Optimal Bandwidths Search History

Iterations	Intercept	TOTALPOPUL	LACK_HEALT	PHYSICAL_I	FREQUENT_P	DRUGS	PVTY_AGE	UNEMP	AICc
0	50	50	50	50	50	50	50	50	827.6
1	30	50	33	39	50	48	50	50	829.5
2	30	50	33	39	50	49	50	50	801.5
3	30	50	34	42	50	50	50	50	797
4	30	50	34	43	50	50	50	50	795.1
5	30	50	34	48	50	50	50	50	794
6	30	50	38	48	50	50	50	50	791.8

Bandwidth Statistics Summary

Explanatory Variables	Optimal Number of Neighbors	Effective Number of Parameters	Adjusted Value of Alpha	Adjusted Critical Value of Pseudo-t Statistics
Intercept	30	28.85	0.0017	3.1577
TOTALPOPUL	50	18.68	0.0027	3.025
LACK_HEALT	38	23.03	0.0022	3.0894
PHYSICAL_I	48	16.75	0.003	2.991
FREQUENT_P	50	17.11	0.0029	2.9976
DRUGS	50	17.35	0.0029	3.0019
PVTY_AGE	50	19.2	0.0026	3.0335
UNEMP	50	17.99	0.0028	3.0133

S2: Summary Statistics for Coefficients Estimates

Explanatory Variables	Mean	Standard Deviation	Minimum	Median	Maximum
Intercept	-0.09	0.49	-1.2907	-0.0806	1.0211
TOTALPOPUL	0.04	0.10	-0.3039	0.0383	0.4011
LACK_HEALT	0.61	0.47	-0.4361	0.5369	2.7777
PHYSICAL_I	-0.18	0.27	-0.9839	-0.1813	0.4115
FREQUENT_P	0.20	0.22	-0.5789	0.1928	0.7358
DRUGS	-0.38	0.23	-0.899	-0.3368	0.288
PVTY_AGE	0.10	0.16	-0.586	0.125	0.5408
UNEMP	0.03	0.17	-0.594	0.0301	0.5437

Model Diagnostics

Statistic	MGWR
R-Squared	0.9098
Adjusted R-Squared	0.8693
AICc	2437.7405
Sigma-Squared	0.1307
Sigma-Squared MLE	0.0902
Effective Degrees of Freedom	1261.3482

Summary of Explanatory Variables and Neighborhoods

Explanatory Variables	Neighbors (% of Features)[a]	Significant (% of Features)[b]
Intercept	30 (1.64)	617 (33.75)
TOTALPOPUL	50 (2.74)	87 (4.76)
LACK_HEALT	50 (2.74)	1156 (63.24)
PHYSICAL_I	45 (2.46)	405 (22.16)
FREQUENT_P	50 (2.74)	435 (23.80)
DRUGS	45 (2.46)	656 (35.89)
PVTY_AGE	49 (2.68)	78 (4.27)
UNEMP	50 (2.74)	39 (2.13)

a: This number in the parenthesis ranges from 0 to 100%, and can be interpreted as a local, regional, global scale based on the geographical context from low to high.

b: In the parentheses, the percentage of features that have significant coefficients of an explanatory variable.

Optimal Bandwidths Search History

Iterations	Intercept	TOTALPOPUL	LACK_HEALT	PHYSICAL_I	FREQUENT_P	DRUGS	PVTY_AGE	UNEMP	AICc
1	30	50	50	50	50	50	50	50	2865
2	30	50	50	50	50	49	50	50	2693
3	30	50	50	50	50	50	50	50	2620
4	30	50	50	50	50	50	50	50	2574
5	30	50	50	50	50	50	50	50	2541
6	30	50	50	50	50	45	50	50	2518
7	30	50	50	50	50	45	49	50	2499
8	30	50	50	50	50	45	49	50	2483
9	30	50	50	50	50	45	49	50	2472
10	30	50	50	50	50	45	49	50	2463
11	30	50	50	50	50	45	49	50	2456
12	30	50	50	50	50	45	49	50	2450
13	30	50	50	50	50	45	49	50	2446
14	30	50	50	50	50	45	49	50	2443
15	30	50	50	50	50	45	49	50	2441
16	30	50	50	45	50	45	49	50	2438

Bandwidth Statistics Summary

Explanatory Variables	Optimal Number of Neighbors	Effective Number of Parameters	Adjusted Value of Alpha	Adjusted Critical Value of Pseudo-t Statistics
Intercept	30	120.05	0.0004	3.5388
TOTALPOPUL	50	72.23	0.0007	3.4011
LACK_HEALT	50	50.28	0.001	3.2998
PHYSICAL_I	45	56.78	0.0009	3.3341
FREQUENT_P	50	53.63	0.0009	3.318
DRUGS	45	74.16	0.0007	3.4083
PVTY_AGE	49	70.26	0.0007	3.3934
UNEMP	50	69.27	0.0007	3.3895

S3: Summary Statistics for Coefficients Estimates

Explanatory Variables	Mean	Standard Deviation	Minimum	Median	Maximum
Intercept	0.15	0.33	-0.405	0.0571	0.8565
TOTALPOPUL	-0.01	0.12	-0.2113	0.0024	0.2299
LACK_HEALT	0.27	0.26	-0.1392	0.2331	0.9842
PHYSICAL_I	0.06	0.39	-0.9158	0.0875	0.8078
FREQUENT_P	0.03	0.39	-0.568	-0.0062	0.7426
DRUGS	-0.51	0.17	-0.8028	-0.5411	-0.107
PVTY_AGE	0.13	0.13	-0.0646	0.1037	0.5189
UNEMP	0.06	0.11	-0.3374	0.031	0.3573

Model Diagnostics

Statistic	GWR	MGWR
R-Squared	0.8637	0.8736
Adjusted R-Squared	0.7762	0.8153
AICc	795.207	745.3683
Sigma-Squared	0.2235	0.1845
Sigma-Squared MLE	0.1363	0.1264
Effective Degrees of Freedom	267.1152	300.0357

Optimal GWR Bandwidth: 50 (K nearest neighbors).

Summary of Explanatory Variables and Neighborhoods

Explanatory Variables	Neighbors (% of Features)[a]	Significant (% of Features)[b]
Intercept	33 (7.53)	85 (19.41)
TOTALPOPUL	50 (11.42)	0 (0.00)
LACK_HEALT	50 (11.42)	14 (3.20)
PHYSICAL_I	44 (10.05)	97 (22.15)
FREQUENT_P	47 (10.73)	157 (35.84)
DRUGS	50 (11.42)	304 (69.41)
PVTY_AGE	50 (11.42)	53 (12.10)
UNEMP	50 (11.42)	0 (0.00)

a: This number in the parenthesis ranges from 0 to 100%, and can be interpreted as a local, regional, global scale based on the geographical context from low to high.

b: In the parentheses, the percentage of features that have significant coefficients of an explanatory variable.

Optimal Bandwidths Search History

Iterations	Intercept	TOTALPOPUL	LACK_HEALT	PHYSICAL_I	FREQUENT_P	DRUGS	PVTY_AGE	UNEMP	AICc
0	50	50	50	50	50	50	50	50	795.2
1	33	50	47	41	47	50	50	50	764.4
2	33	50	47	44	47	50	50	50	750.6
3	33	50	50	44	47	50	50	50	745.4

Bandwidth Statistics Summary

Explanatory Variables	Optimal Number of Neighbors	Effective Number of Parameters	Adjusted Value of Alpha	Adjusted Critical Value of Pseudo-t Statistics
Intercept	33	24.69	0.002	3.1138
TOTALPOPUL	50	16.76	0.003	2.9939
LACK_HEALT	50	15.07	0.0033	2.9604
PHYSICAL_I	44	17.57	0.0028	3.0087
FREQUENT_P	47	15.79	0.0032	2.9752
DRUGS	50	13.13	0.0038	2.9165
PVTY_AGE	50	16.58	0.003	2.9905
UNEMP	50	18.38	0.0027	3.0227

S4: Summary Statistics for Coefficients Estimates

Explanatory Variables	Mean	Standard Deviation	Minimum	Median	Maximum
Intercept	0.23	0.33	-0.418	0.177	1.0808
TOTALPOPUL	0.00	0.17	-0.3699	-0.0322	0.3274
LACK_HEALT	0.49	0.45	-0.1225	0.3199	1.6074
PHYSICAL_I	0.17	0.46	-0.6688	0.2908	0.9457
FREQUENT_P	-0.37	0.60	-1.1439	-0.289	0.8302
DRUGS	-0.13	0.45	-0.88	-0.065	0.6182
PVTY_AGE	0.27	0.40	-0.3329	0.1846	0.8547
UNEMP	0.06	0.19	-0.1232	-0.0034	0.58

Model Diagnostics

Statistic	GWR	MGWR
R-Squared	0.8381	0.8652
Adjusted R-Squared	0.7273	0.7916
AICc	531.9183	503.8539
Sigma-Squared	0.2719	0.208
Sigma-Squared MLE	0.1619	0.1348
Effective Degrees of Freedom	154.1757	167.823

Optimal GWR Bandwidth: 50 (K nearest neighbors).

Summary of Explanatory Variables and Neighborhoods

Explanatory Variables	Neighbors (% of Features)[a]	Significant (% of Features)[b]
Intercept	30 (11.58)	44 (16.99)
TOTALPOPUL	50 (19.31)	31 (11.97)
LACK_HEALT	35 (13.51)	67 (25.87)
PHYSICAL_I	50 (19.31)	42 (16.22)
FREQUENT_P	34 (13.13)	128 (49.42)
DRUGS	50 (19.31)	137 (52.90)
PVTY_AGE	39 (15.06)	66 (25.48)
UNEMP	50 (19.31)	34 (13.13)

a: This number in the parenthesis ranges from 0 to 100%, and can be interpreted as a local, regional, global scale based on the geographical context from low to high.

b: In the parentheses, the percentage of features that have significant coefficients of an explanatory variable.

Optimal Bandwidths Search History

Iterations	Intercept	TOTALPOPUL	LACK_HEALT	PHYSICAL_I	FREQUENT_P	DRUGS	PVTY_AGE	UNEMP	AICc
0	50	50	50	50	50	50	50	50	531.9
1	31	50	35	43	32	50	39	42	526.3
2	31	50	35	47	33	50	39	50	506.5
3	30	50	35	50	34	50	39	50	503.9

Bandwidth Statistics Summary

Explanatory Variables	Optimal Number of Neighbors	Effective Number of Parameters	Adjusted Value of Alpha	Adjusted Critical Value of Pseudo-t Statistics
Intercept	30	14.43	0.0035	2.9652
TOTALPOPUL	50	10.23	0.0049	2.8524
LACK_HEALT	35	14.34	0.0035	2.9633
PHYSICAL_I	50	9.73	0.0051	2.8355
FREQUENT_P	34	12.76	0.0039	2.9252
DRUGS	50	9.05	0.0055	2.8112
PVTY_AGE	39	10.46	0.0048	2.8597
UNEMP	50	10.17	0.0049	2.8501

C1: Summary Statistics for Coefficients Estimates

Explanatory Variables	Mean	Standard Deviation	Minimum	Median	Maximum
Intercept	0.05	0.10	-0.1044	0.0163	0.2021
TOTALPOPUL	-0.01	0.08	-0.1296	-0.0364	0.1044
LACK_HEALT	0.29	0.11	0.0197	0.3068	0.4082
PHYSICAL_I	-0.58	0.13	-0.725	-0.6108	-0.3487
FREQUENT_P	0.93	0.23	0.6889	0.8029	1.385
DRUGS	-0.85	0.19	-1.1258	-0.8067	-0.5005
PVTY_AGE	-0.07	0.04	-0.1292	-0.0465	-0.0014
UNEMP	0.02	0.19	-0.141	-0.0769	0.4533

Model Diagnostics

Statistic	GWR	MGWR
R-Squared	0.8221	0.8617
Adjusted R-Squared	0.7158	0.794
AICc	167.3478	151.6801
Sigma-Squared	0.2821	0.2047
Sigma-Squared MLE	0.1779	0.1383
Effective Degrees of Freedom	50.4537	54.0294

Optimal GWR Bandwidth: 48 (K nearest neighbors).

Summary of Explanatory Variables and Neighborhoods

Explanatory Variables	Neighbors (% of Features)[a]	Significant (% of Features)[b]
Intercept	50 (62.50)	0 (0.00)
TOTALPOPUL	45 (56.25)	0 (0.00)
LACK_HEALT	50 (62.50)	22 (27.50)
PHYSICAL_I	45 (56.25)	50 (62.50)
FREQUENT_P	30 (37.50)	80 (100.00)
DRUGS	31 (38.75)	74 (92.50)
PVTY_AGE	50 (62.50)	0 (0.00)
UNEMP	33 (41.25)	19 (23.75)

a: This number in the parenthesis ranges from 0 to 100%, and can be interpreted as a local, regional, global scale based on the geographical context from low to high.

b: In the parentheses, the percentage of features that have significant coefficients of an explanatory variable.

Optimal Bandwidths Search History

Iterations	Intercept	TOTALPOPUL	LACK_HEALT	PHYSICAL_I	FREQUENT_P	DRUGS	PVTY_AGE	UNEMP	AICc
0	48	48	48	48	48	48	48	48	167.3
1	50	48	30	45	30	50	50	38	160.2
2	50	45	30	45	30	50	50	32	155.4
3	50	45	50	45	30	30	50	33	151.9
4	50	45	50	45	30	31	50	33	151.7

Bandwidth Statistics Summary

Explanatory Variables	Optimal Number of Neighbors	Effective Number of Parameters	Adjusted Value of Alpha	Adjusted Critical Value of Pseudo-t Statistics
Intercept	50	2.86	0.0175	2.4524
TOTALPOPUL	45	3.43	0.0146	2.5243
LACK_HEALT	50	3.13	0.016	2.4871
PHYSICAL_I	45	2.76	0.0181	2.4378
FREQUENT_P	30	4.22	0.0119	2.6047
DRUGS	31	3.5	0.0143	2.5314
PVTY_AGE	50	2.55	0.0196	2.4052
UNEMP	33	3.52	0.0142	2.5342

C2: Summary Statistics for Coefficients Estimates

Explanatory Variables	Mean	Standard Deviation	Minimum	Median	Maximum
Intercept	0.08	0.39	-0.7886	0.0112	1.2809
TOTALPOPUL	0.04	0.10	-0.232	0.0335	0.4865
LACK_HEALT	0.20	0.37	-0.7114	0.1726	0.993
PHYSICAL_I	-0.06	0.43	-1.2058	0.1139	0.5804
FREQUENT_P	0.10	0.37	-0.6911	0.1285	1.019
DRUGS	-0.62	0.26	-1.2902	-0.6038	0.2089
PVTY_AGE	0.34	0.16	-0.1158	0.3552	0.8376
UNEMP	0.04	0.12	-0.2589	0.05	0.3781

Model Diagnostics

Statistic	GWR	MGWR
R-Squared	0.918	0.9288
Adjusted R-Squared	0.8618	0.894
AICc	1651.1393	1440.4363
Sigma-Squared	0.1381	0.1059
Sigma-Squared MLE	0.082	0.0712
Effective Degrees of Freedom	728.3559	824.413

Optimal GWR Bandwidth: 50 (K nearest neighbors).

Summary of Explanatory Variables and Neighborhoods

Explanatory Variables	Neighbors (% of Features)[a]	Significant (% of Features)[b]
Intercept	30 (2.45)	248 (20.23)
TOTALPOPUL	50 (4.08)	47 (3.83)
LACK_HEALT	50 (4.08)	143 (11.66)
PHYSICAL_I	50 (4.08)	152 (12.40)
FREQUENT_P	50 (4.08)	132 (10.77)
DRUGS	32 (2.61)	829 (67.62)
PVTY_AGE	50 (4.08)	715 (58.32)
UNEMP	50 (4.08)	28 (2.28)

a: This number in the parenthesis ranges from 0 to 100%, and can be interpreted as a local, regional, global scale based on the geographical context from low to high.

b: In the parentheses, the percentage of features that have significant coefficients of an explanatory variable.

Optimal Bandwidths Search History

Iterations	Intercept	TOTALPOPUL	LACK_HEALT	PHYSICAL_I	FREQUENT_P	DRUGS	PVTY_AGE	UNEMP	AICc
0	50	50	50	50	50	50	50	50	1651
1	30	50	30	30	31	32	47	50	1696
2	30	50	30	31	38	32	47	50	1573
3	30	50	38	38	45	32	50	50	1511
4	30	50	38	38	50	32	50	50	1491
5	30	50	38	38	50	32	50	50	1480
6	30	50	38	38	50	32	50	50	1474
7	30	50	44	38	50	32	50	50	1467
8	30	50	50	38	50	32	50	50	1463
9	30	50	50	50	50	32	50	50	1449
10	30	50	50	50	50	32	50	50	1444
11	30	50	50	50	50	32	50	50	1442
12	30	50	50	50	50	32	50	50	1440

Bandwidth Statistics Summary

Explanatory Variables	Optimal Number of Neighbors	Effective Number of Parameters	Adjusted Value of Alpha	Adjusted Critical Value of Pseudo-t Statistics
Intercept	30	79.01	0.0006	3.4303
TOTALPOPUL	50	52.43	0.001	3.3158
LACK_HEALT	50	37.9	0.0013	3.2228
PHYSICAL_I	50	38.89	0.0013	3.2303
FREQUENT_P	50	37.33	0.0013	3.2184
DRUGS	32	63.79	0.0008	3.371
PVTY_AGE	50	45.05	0.0011	3.2726
UNEMP	50	47.18	0.0011	3.2859

C3: Summary Statistics for Coefficients Estimates

Explanatory Variables	Mean	Standard Deviation	Minimum	Median	Maximum
Intercept	0.08	0.43	-0.823	0.0469	1.8931
TOTALPOPUL	-0.05	0.16	-0.6711	-0.0433	0.4831
LACK_HEALT	0.16	0.43	-0.6418	0.0316	1.395
PHYSICAL_I	0.21	0.67	-1.1639	0.2375	2.0207
FREQUENT_P	0.08	0.52	-1.2284	0.0691	1.3948
DRUGS	-0.32	0.30	-0.8265	-0.3753	0.562
PVTY_AGE	0.20	0.24	-0.49	0.1702	0.8605
UNEMP	0.05	0.12	-0.3229	0.0624	0.3177

Model Diagnostics

Statistic	GWR	MGWR
R-Squared	0.8706	0.8895
Adjusted R-Squared	0.7831	0.8305
AICc	1290.8426	1225.2677
Sigma-Squared	0.2167	0.1694
Sigma-Squared MLE	0.1294	0.1105
Effective Degrees of Freedom	428.1053	467.9087

Optimal GWR Bandwidth: 49 (K nearest neighbors).

Summary of Explanatory Variables and Neighborhoods

Explanatory Variables	Neighbors (% of Features)[a]	Significant (% of Features)[b]
Intercept	31 (4.32)	114 (15.90)
TOTALPOPUL	50 (6.97)	44 (6.14)
LACK_HEALT	42 (5.86)	31 (4.32)
PHYSICAL_I	39 (5.44)	113 (15.76)
FREQUENT_P	36 (5.02)	101 (14.09)
DRUGS	39 (5.44)	276 (38.49)
PVTY_AGE	42 (5.86)	101 (14.09)
UNEMP	50 (6.97)	31 (4.32)

a: This number in the parenthesis ranges from 0 to 100%, and can be interpreted as a local, regional, global scale based on the geographical context from low to high.

b: In the parentheses, the percentage of features that have significant coefficients of an explanatory variable.

Optimal Bandwidths Search History

Iterations	Intercept	TOTALPOPUL	LACK_HEALT	PHYSICAL_I	FREQUENT_P	DRUGS	PVTY_AGE	UNEMP	AICc
0	49	49	49	49	49	49	49	49	1291
1	30	39	30	30	32	42	43	50	1352
2	30	48	30	33	35	39	42	50	1272
3	31	48	35	39	36	39	43	50	1242
4	31	50	42	39	36	39	42	50	1225

Bandwidth Statistics Summary

Explanatory Variables	Optimal Number of Neighbors	Effective Number of Parameters	Adjusted Value of Alpha	Adjusted Critical Value of Pseudo-t Statistics
Intercept	31	38.82	0.0013	3.2383
TOTALPOPUL	50	26.72	0.0019	3.1278
LACK_HEALT	42	32.11	0.0016	3.1825
PHYSICAL_I	39	32.56	0.0015	3.1867
FREQUENT_P	36	33.35	0.0015	3.1937
DRUGS	39	30.59	0.0016	3.1682
PVTY_AGE	42	29.24	0.0017	3.1547
UNEMP	50	25.71	0.0019	3.1161

T1: Summary Statistics for Coefficients Estimates

Explanatory Variables	Mean	Standard Deviation	Minimum	Median	Maximum
Intercept	0.18	0.59	-1.5055	0.1209	1.9182
TOTALPOPUL	0.00	0.11	-0.3014	-0.0077	0.3435
LACK_HEALT	0.82	0.64	-0.9248	0.6469	2.4826
PHYSICAL_I	-0.01	0.34	-0.8506	0.0161	1.0961
FREQUENT_P	0.12	0.28	-0.5831	0.1332	0.7579
DRUGS	-0.21	0.23	-1.2194	-0.1629	0.4621
PVTY_AGE	0.05	0.13	-0.3027	0.0263	0.5248
UNEMP	0.03	0.10	-0.4953	0.0226	0.6891

Model Diagnostics

Statistic	GWR	MGWR
R-Squared	0.8986	0.9129
Adjusted R-Squared	0.8311	0.8659
AICc	3344.1366	3245.52
Sigma-Squared	0.1688	0.1341
Sigma-Squared MLE	0.1014	0.0871
Effective Degrees of Freedom	1316.8864	1422.88

Optimal GWR Bandwidth: 50 (K nearest neighbors).

Summary of Explanatory Variables and Neighborhoods

Explanatory Variables	Neighbors (% of Features)[a]	Significant (% of Features)[b]
Intercept	30 (1.37)	700 (31.93)
TOTALPOPUL	50 (2.28)	19 (0.87)
LACK_HEALT	37 (1.69)	917 (41.83)
PHYSICAL_I	50 (2.28)	104 (4.74)
FREQUENT_P	35 (1.60)	133 (6.07)
DRUGS	31 (1.41)	318 (14.51)
PVTY_AGE	50 (2.28)	68 (3.10)
UNEMP	50 (2.28)	39 (1.78)

a: This number in the parenthesis ranges from 0 to 100%, and can be interpreted as a local, regional, global scale based on the geographical context from low to high.

b: In the parentheses, the percentage of features that have significant coefficients of an explanatory variable.

Optimal Bandwidths Search History

Iterations	Intercept	TOTALPOPUL	LACK_HEALT	PHYSICAL_I	FREQUENT_P	DRUGS	PVTY_AGE	UNEMP	AICc
0	50	50	50	50	50	50	50	50	3344
1	30	50	30	30	31	43	50	50	3519
2	30	50	30	30	34	43	50	50	3358
3	30	50	30	30	35	43	50	50	3319
4	30	50	34	30	35	43	50	50	3295
5	30	50	35	34	35	37	50	50	3284
6	30	50	35	45	35	34	50	50	3269
7	30	50	35	50	35	31	50	50	3265
8	30	50	35	50	35	31	50	50	3258
9	30	50	36	50	35	31	50	50	3253
10	30	50	36	50	35	31	50	50	3250
11	30	50	36	50	35	31	50	50	3249
12	30	50	37	50	35	31	50	50	3247
13	30	50	37	50	35	31	50	50	3246
14	30	50	37	50	35	31	50	50	3246
15	30	50	37	50	35	31	50	50	3246

Bandwidth Statistics Summary

Explanatory Variables	Optimal Number of Neighbors	Effective Number of Parameters	Adjusted Value of Alpha	Adjusted Critical Value of Pseudo-t Statistics
Intercept	30	126.49	0.0004	3.5517
TOTALPOPUL	50	86.66	0.0006	3.45
LACK_HEALT	37	92.14	0.0005	3.4667
PHYSICAL_I	50	58.63	0.0009	3.3422
FREQUENT_P	35	104.78	0.0005	3.5014
DRUGS	31	133.22	0.0004	3.5654
PVTY_AGE	50	82.69	0.0006	3.4372
UNEMP	50	84.52	0.0006	3.4432

T2: Summary Statistics for Coefficients Estimates

Explanatory Variables	Mean	Standard Deviation	Minimum	Median	Maximum
Intercept	0.22	0.81	-2.0195	0.1618	2.6403
TOTALPOPUL	0.06	0.19	-0.6826	0.0366	1.2062
LACK_HEALT	1.03	0.64	-0.8422	1.0579	2.8802
PHYSICAL_I	-0.08	0.45	-1.4382	-0.0839	2.1271
FREQUENT_P	0.06	0.31	-1.2115	0.0421	1.0676
DRUGS	-0.02	0.13	-1.2359	-0.0156	0.469
PVTY_AGE	0.02	0.09	-0.2847	0.0148	0.4231
UNEMP	0.01	0.09	-0.3399	0.0054	0.616

Model Diagnostics

Statistic	GWR	MGWR
R-Squared	0.9434	0.9514
Adjusted R-Squared	0.9016	0.9225
AICc	6083.0738	6091.4206
Sigma-Squared	0.0984	0.0775
Sigma-Squared MLE	0.0566	0.0486
Effective Degrees of Freedom	3497.3702	3812.5287

Optimal GWR Bandwidth: 50 (K nearest neighbors).

Summary of Explanatory Variables and Neighborhoods

Explanatory Variables	Neighbors (% of Features)[a]	Significant (% of Features)[b]
Intercept	30 (0.49)	2120 (34.89)
TOTALPOPUL	50 (0.82)	57 (0.94)
LACK_HEALT	32 (0.53)	2582 (42.50)
PHYSICAL_I	33 (0.54)	412 (6.78)
FREQUENT_P	34 (0.56)	347 (5.71)
DRUGS	35 (0.58)	76 (1.25)
PVTY_AGE	50 (0.82)	60 (0.99)
UNEMP	50 (0.82)	63 (1.04)

a: This number in the parenthesis ranges from 0 to 100%, and can be interpreted as a local, regional, global scale based on the geographical context from low to high.

b: In the parentheses, the percentage of features that have significant coefficients of an explanatory variable.

Optimal Bandwidths Search History

Iterations	Intercept	TOTALPOPUL	LACK_HEALT	PHYSICAL_I	FREQUENT_P	DRUGS	PVTY_AGE	UNEMP	AICc
0	50	50	50	50	50	50	50	50	6083
1	30	37	30	30	30	41	50	50	7260
2	30	41	30	30	31	36	50	50	6564
3	30	50	30	30	32	35	50	50	6299
4	30	50	31	31	34	35	50	50	6167
5	30	50	32	33	34	35	50	50	6091

Bandwidth Statistics Summary

Explanatory Variables	Optimal Number of Neighbors	Effective Number of Parameters	Adjusted Value of Alpha	Adjusted Critical Value of Pseudo-t Statistics
Intercept	30	323.23	0.0002	3.7872
TOTALPOPUL	50	206.78	0.0002	3.6743
LACK_HEALT	32	298.6	0.0002	3.7674
PHYSICAL_I	33	297.15	0.0002	3.7662
FREQUENT_P	34	293.97	0.0002	3.7635
DRUGS	35	351.13	0.0001	3.8078
PVTY_AGE	50	244.5	0.0002	3.717
UNEMP	50	248.1	0.0002	3.7207

T3: Summary Statistics for Coefficients Estimates

Explanatory Variables	Mean	Standard Deviation	Minimum	Median	Maximum
Intercept	0.28	0.93	-2.7586	0.0871	2.9417
TOTALPOPUL	0.05	0.13	-0.4203	0.0345	0.5143
LACK_HEALT	1.11	0.74	-1.4147	1.0448	3.6423
PHYSICAL_I	-0.23	0.56	-3.5351	-0.2062	1.843
FREQUENT_P	0.19	0.40	-1.2605	0.1675	2.2777
DRUGS	-0.02	0.13	-0.6584	-0.0081	0.4739
PVTY_AGE	0.04	0.09	-0.3473	0.0321	0.6045
UNEMP	0.04	0.10	-0.2931	0.0331	0.5575

Model Diagnostics

Statistic	GWR	MGWR
R-Squared	0.9351	0.9421
Adjusted R-Squared	0.8866	0.9099
AICc	8113.262	7777.2422
Sigma-Squared	0.1134	0.0901
Sigma-Squared MLE	0.0649	0.0579
Effective Degrees of Freedom	4037.9784	4533.2887

Optimal GWR Bandwidth: 50 (K nearest neighbors).

Summary of Explanatory Variables and Neighborhoods

Explanatory Variables	Neighbors (% of Features)[a]	Significant (% of Features)[b]
Intercept	30 (0.43)	2653 (37.60)
TOTALPOPUL	50 (0.71)	12 (0.17)
LACK_HEALT	30 (0.43)	2525 (35.79)
PHYSICAL_I	47 (0.67)	832 (11.79)
FREQUENT_P	34 (0.48)	652 (9.24)
DRUGS	47 (0.67)	70 (0.99)
PVTY_AGE	50 (0.71)	39 (0.55)
UNEMP	50 (0.71)	87 (1.23)

a: This number in the parenthesis ranges from 0 to 100%, and can be interpreted as a local, regional, global scale based on the geographical context from low to high.

b: In the parentheses, the percentage of features that have significant coefficients of an explanatory variable.

Optimal Bandwidths Search History

Iterations	Intercept	TOTALPOPUL	LACK_HEALT	PHYSICAL_I	FREQUENT_P	DRUGS	PVTY_AGE	UNEMP	AICc
0	50	50	50	50	50	50	50	50	8113.262
1	30	47	30	30	30	49	50	50	9527.325
2	30	50	30	30	30	47	50	50	8488.045
3	30	50	30	30	31	47	50	50	8212.412
4	30	50	30	31	31	47	50	50	8077.249
5	30	50	30	35	31	47	50	50	7983.217
6	30	50	30	39	30	47	50	50	7927.762
7	30	50	30	42	31	47	50	50	7868.183
8	30	50	30	44	31	47	50	50	7842.309
9	30	50	30	45	34	47	50	50	7791.662
10	30	50	30	47	34	47	50	50	7777.242

Bandwidth Statistics Summary

Explanatory Variables	Optimal Number of Neighbors	Effective Number of Parameters	Adjusted Value of Alpha	Adjusted Critical Value of Pseudo-t Statistics
Intercept	30	391.1	0.0001	3.8339
TOTALPOPUL	50	258.25	0.0002	3.7303
LACK_HEALT	30	388.64	0.0001	3.8323
PHYSICAL_I	47	224.88	0.0002	3.6952
FREQUENT_P	34	369.15	0.0001	3.8196
DRUGS	47	309.55	0.0002	3.7758
PVTY_AGE	50	286.53	0.0002	3.7565
UNEMP	50	294.61	0.0002	3.7634

T4: Summary Statistics for Coefficients Estimates

Explanatory Variables	Mean	Standard Deviation	Minimum	Median	Maximum
Intercept	0.21	1.06	-2.8705	0.0893	3.5948
TOTALPOPUL	0.04	0.13	-0.5057	0.0333	0.7059
LACK_HEALT	0.84	0.66	-1.0755	0.7385	3.3034
PHYSICAL_I	-0.12	0.51	-1.8222	-0.1143	2.099
FREQUENT_P	0.23	0.42	-1.0426	0.2323	1.8799
DRUGS	-0.05	0.16	-0.9874	-0.0291	0.6811
PVTY_AGE	0.06	0.11	-0.4068	0.0423	0.9914
UNEMP	0.03	0.11	-0.2897	0.0091	0.758

Model Diagnostics

Statistic	GWR	MGWR
R-Squared	0.9326	0.9385
Adjusted R-Squared	0.8809	0.9027
AICc	8991.4105	9097.9518
Sigma-Squared	0.1191	0.0973
Sigma-Squared MLE	0.0674	0.0615
Effective Degrees of Freedom	4243.0193	4737.9561

Optimal GWR Bandwidth: 50 (K nearest neighbors).

Summary of Explanatory Variables and Neighborhoods

Explanatory Variables	Neighbors (% of Features)[a]	Significant (% of Features)[b]
Intercept	32 (0.43)	1541 (20.56)
TOTALPOPUL	50 (0.67)	92 (1.23)
LACK_HEALT	34 (0.45)	1078 (14.38)
PHYSICAL_I	33 (0.44)	329 (4.39)
FREQUENT_P	36 (0.48)	414 (5.52)
DRUGS	38 (0.51)	162 (2.16)
PVTY_AGE	50 (0.67)	97 (1.29)
UNEMP	50 (0.67)	125 (1.67)

a: This number in the parenthesis ranges from 0 to 100%, and can be interpreted as a local, regional, global scale based on the geographical context from low to high.

b: In the parentheses, the percentage of features that have significant coefficients of an explanatory variable.

Optimal Bandwidths Search History

Iterations	Intercept	TOTALPOPUL	LACK_HEALT	PHYSICAL_I	FREQUENT_P	DRUGS	PVTY_AGE	UNEMP	AICc
0	50	50	50	50	50	50	50	50	8991.411
1	30	50	30	30	30	47	50	50	10741.03
2	30	48	30	30	30	46	50	50	9688.996
3	30	50	30	30	30	42	50	50	9416.649
4	30	50	32	30	34	39	50	50	9243.102
5	32	50	34	33	36	38	50	50	9097.952

Bandwidth Statistics Summary

Explanatory Variables	Optimal Number of Neighbors	Effective Number of Parameters	Adjusted Value of Alpha	Adjusted Critical Value of Pseudo-t Statistics
Intercept	32	394.19	0.0001	3.8357
TOTALPOPUL	50	304.59	0.0002	3.7716
LACK_HEALT	34	362.96	0.0001	3.8153
PHYSICAL_I	33	375.54	0.0001	3.8237
FREQUENT_P	36	337.03	0.0001	3.7969
DRUGS	38	387.48	0.0001	3.8314
PVTY_AGE	50	291.65	0.0002	3.7608
UNEMP	50	303.6	0.0002	3.7708

Moran's I Statistic for Prevalence of mental Health: ZCTA-level (left panel) and County-level (right panel)

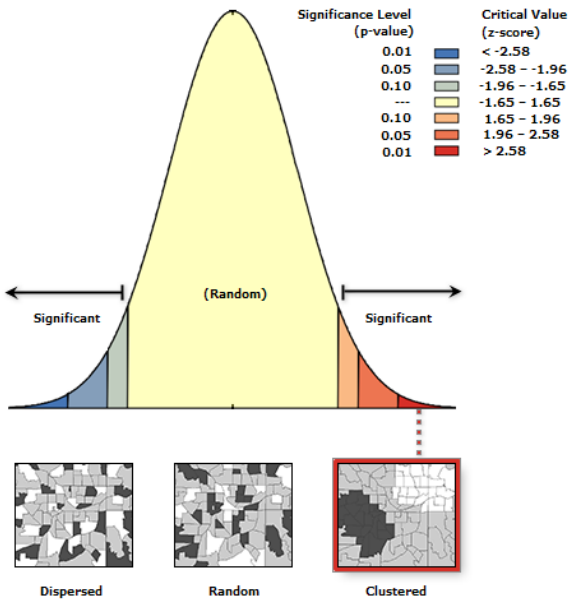

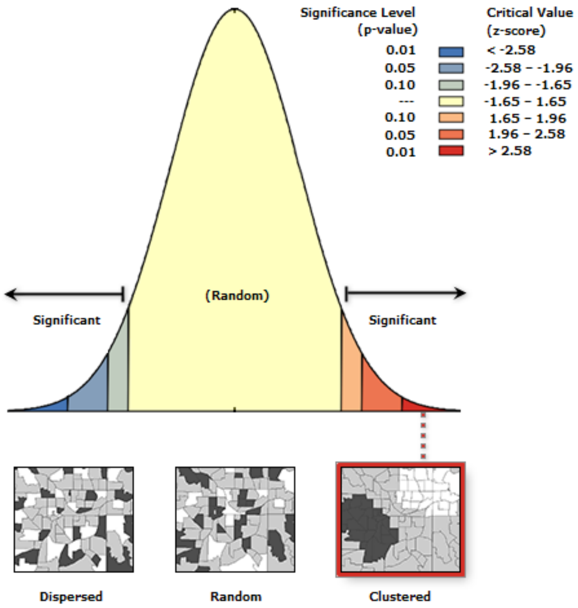

The Moran's I statistic for mental health at the ZCTA-level is 0.56 with a z-scoreof 624, while for mental health at the county-level are 0.70 with z-score of 121. In both models, there is a less than 1% chance that the clustered patters are a result of chance. The following maps visually illustrate this point in which despite of aggregations mental health at both levels display remarkably similar spatial patterns.

Hot Spot Analysis for Crude Prevalence of Mental Health Not Good for > 14 days among Adults 18 Years or Older for ZCTAs (left) and Counties (right)

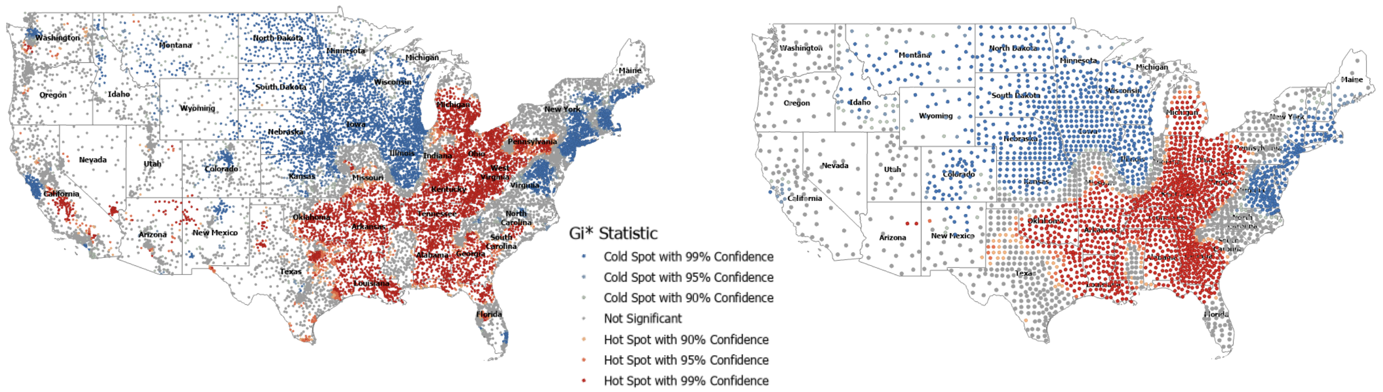

Supplement: Supplementary file 1 [file ijerph-20-05440-s001.zip › Supplementary Files and Appendix A/Appendix A.pdf]
