# Supplementary figures and images for: The Geography of Mental Health, Urbanicity, and Affluence
Source: Int J Environ Res Public Health. 2023 Apr 7;20(8):5440. doi: 10.3390/ijerph20085440 (PMC10138034; doi:10.3390/ijerph20085440)

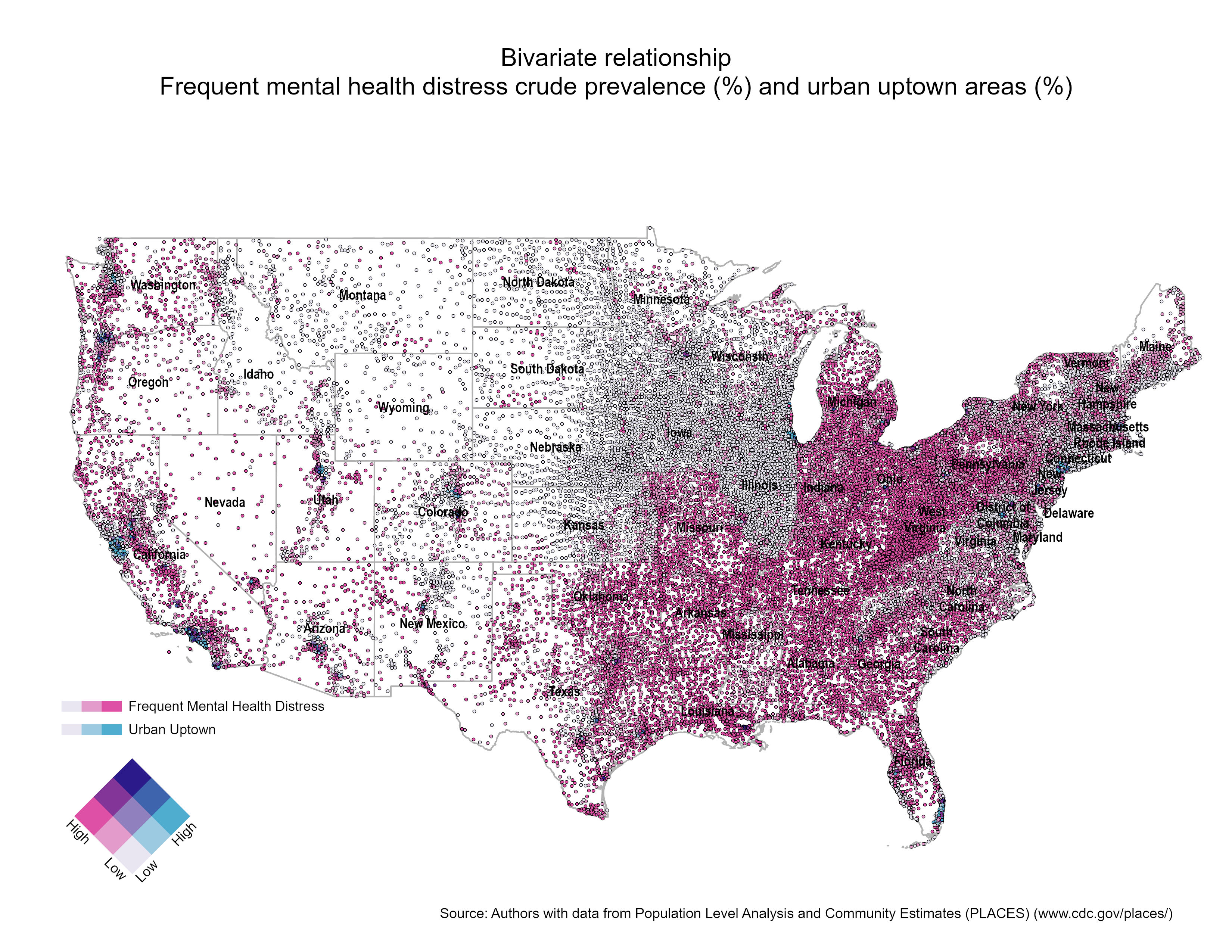

Supplement: Supplementary file 1 [file ijerph-20-05440-s001.zip › Supplementary Files and Appendix A/Figure 2A Urban Uptown.jpg]

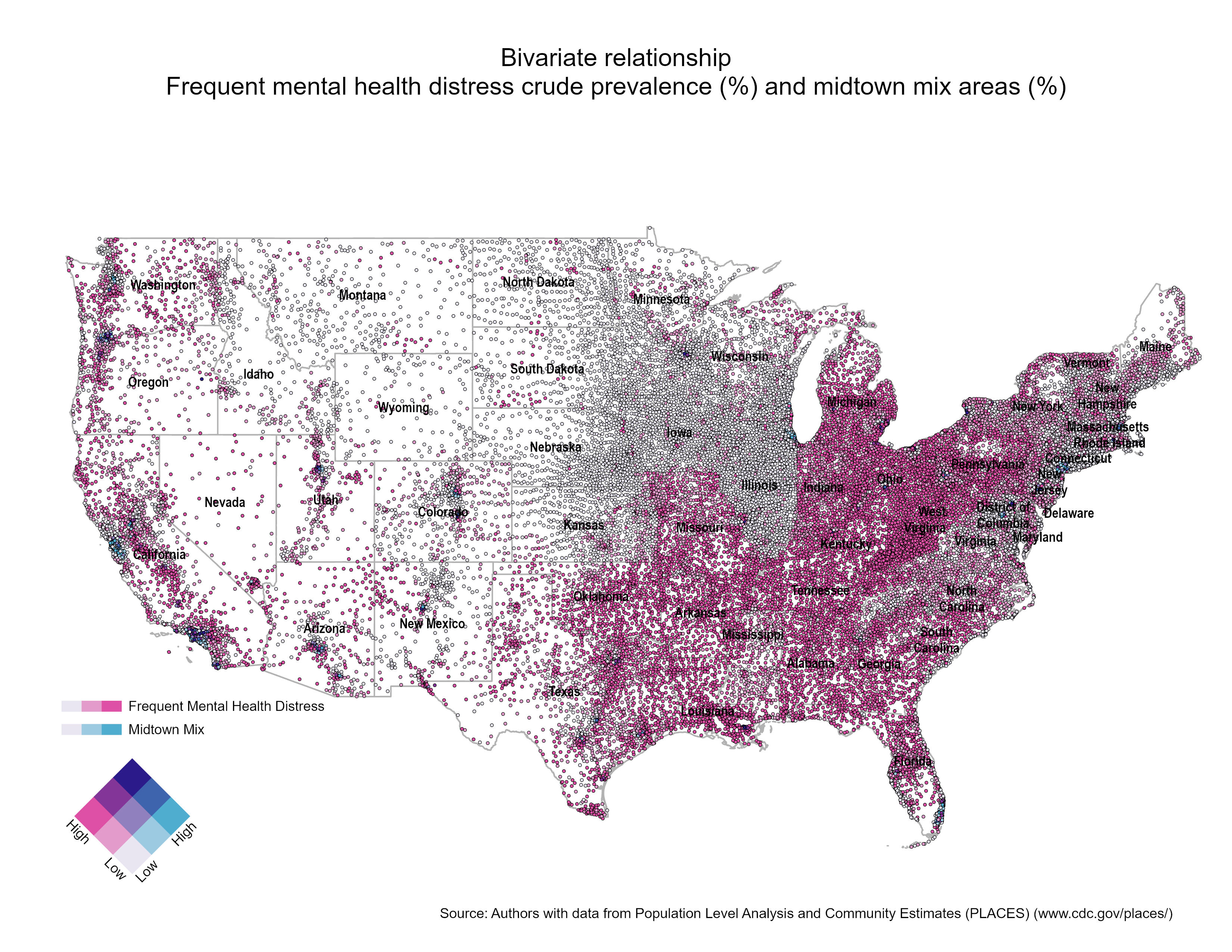

Supplement: Supplementary file 1 [file ijerph-20-05440-s001.zip › Supplementary Files and Appendix A/Figure 2B Midtown Mix.jpg]

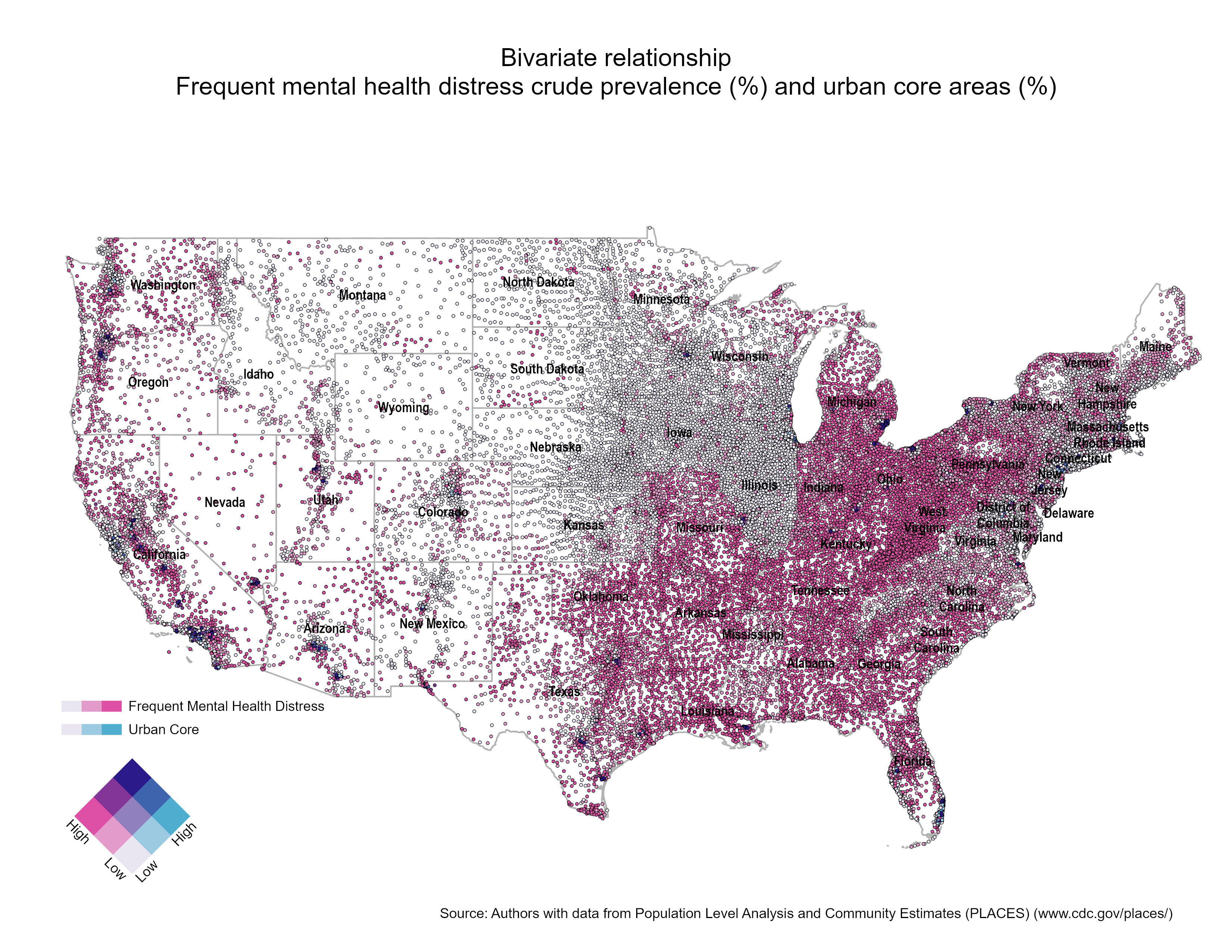

Supplement: Supplementary file 1 [file ijerph-20-05440-s001.zip › Supplementary Files and Appendix A/Figure 2C Urban Core.jpg]

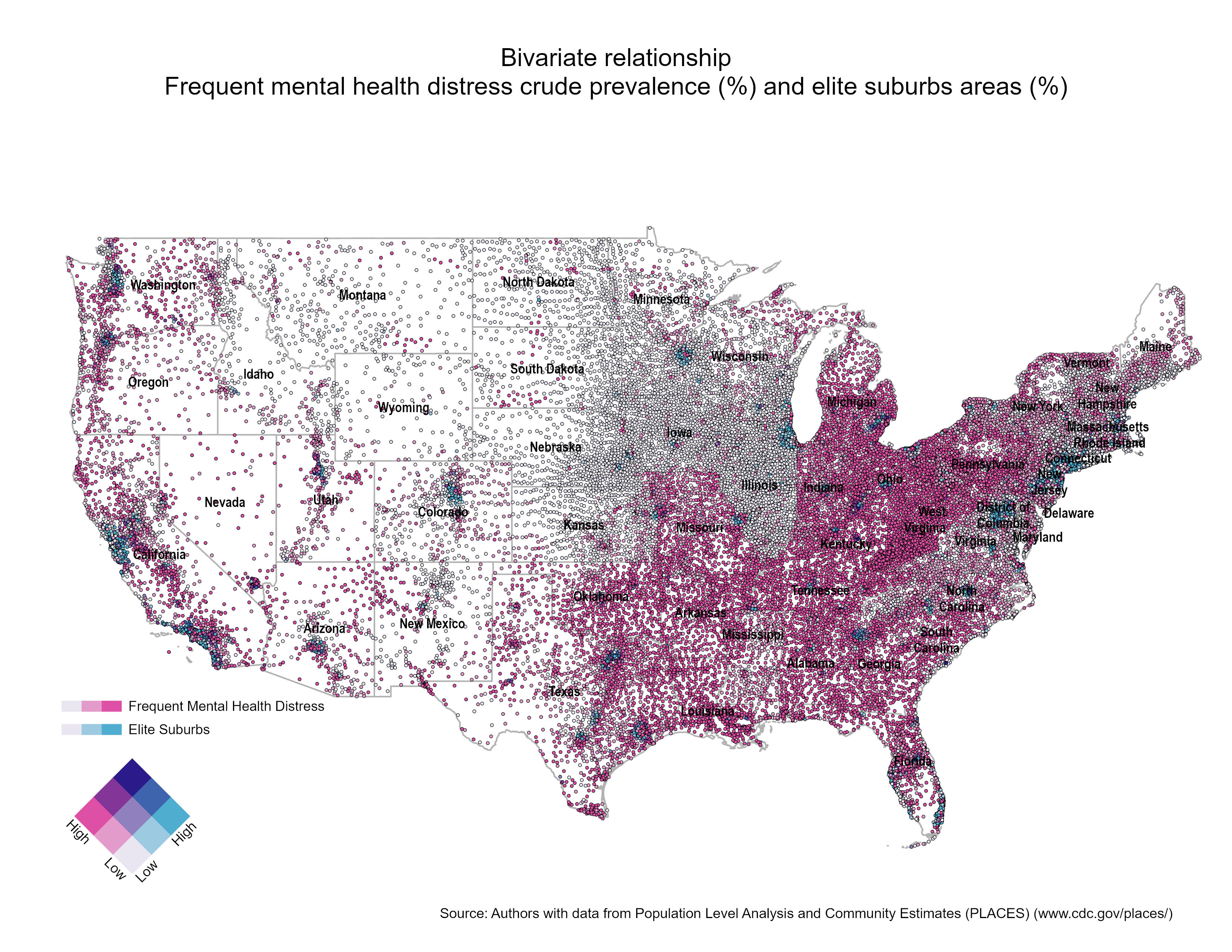

Supplement: Supplementary file 1 [file ijerph-20-05440-s001.zip › Supplementary Files and Appendix A/Figure 2D Elite Suburbs.jpg]

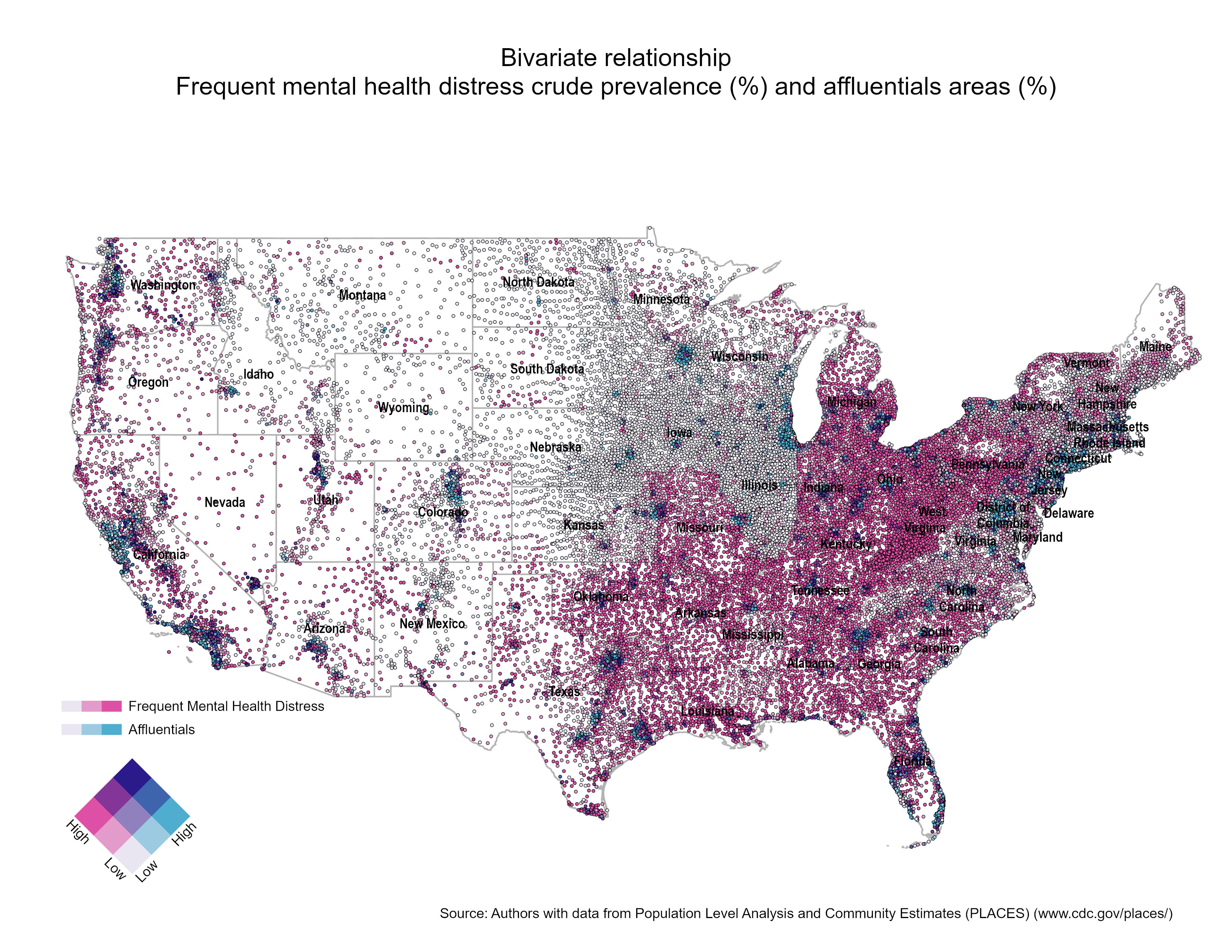

Supplement: Supplementary file 1 [file ijerph-20-05440-s001.zip › Supplementary Files and Appendix A/Figure 2E Affluentials.jpg]

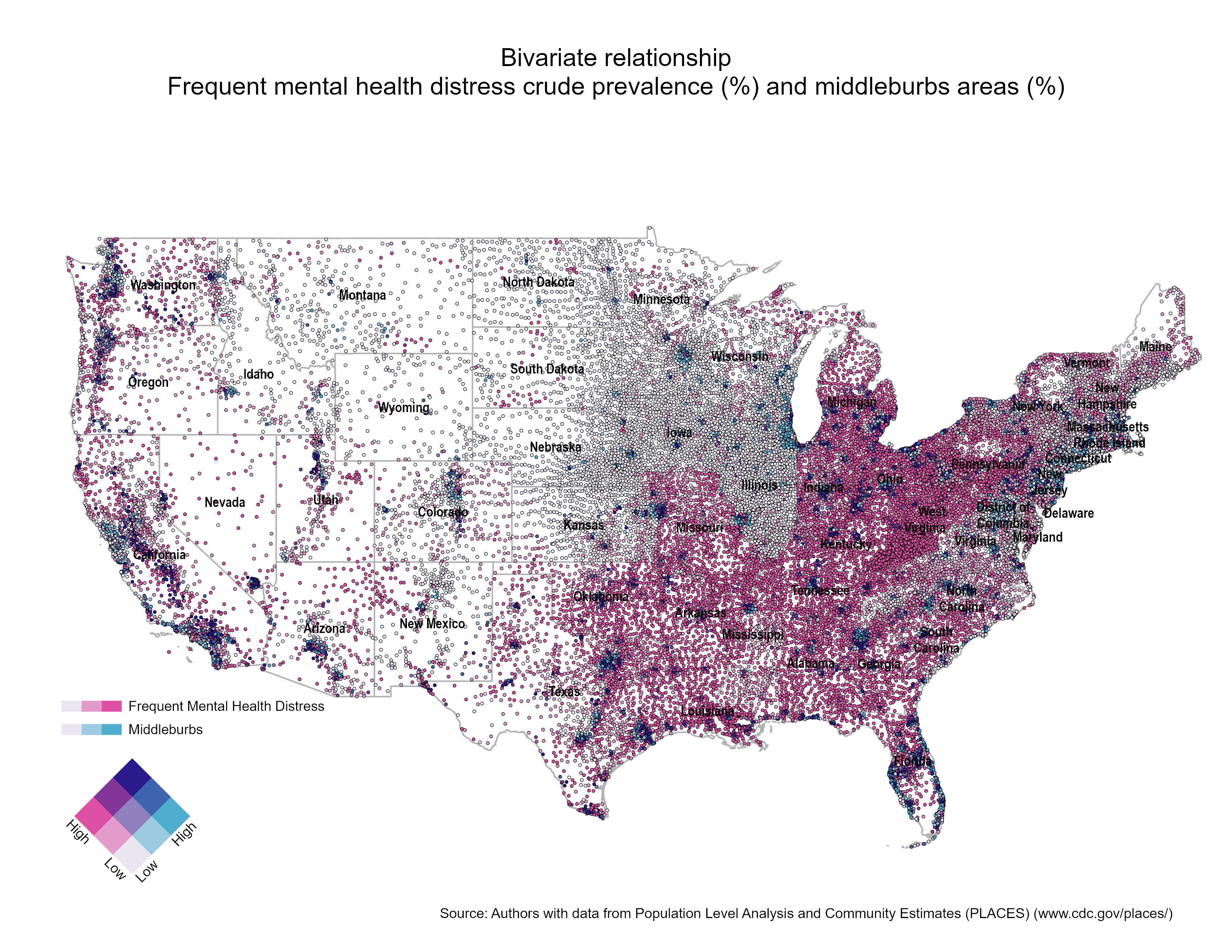

Supplement: Supplementary file 1 [file ijerph-20-05440-s001.zip › Supplementary Files and Appendix A/Figure 2F Middleburbs.jpg]

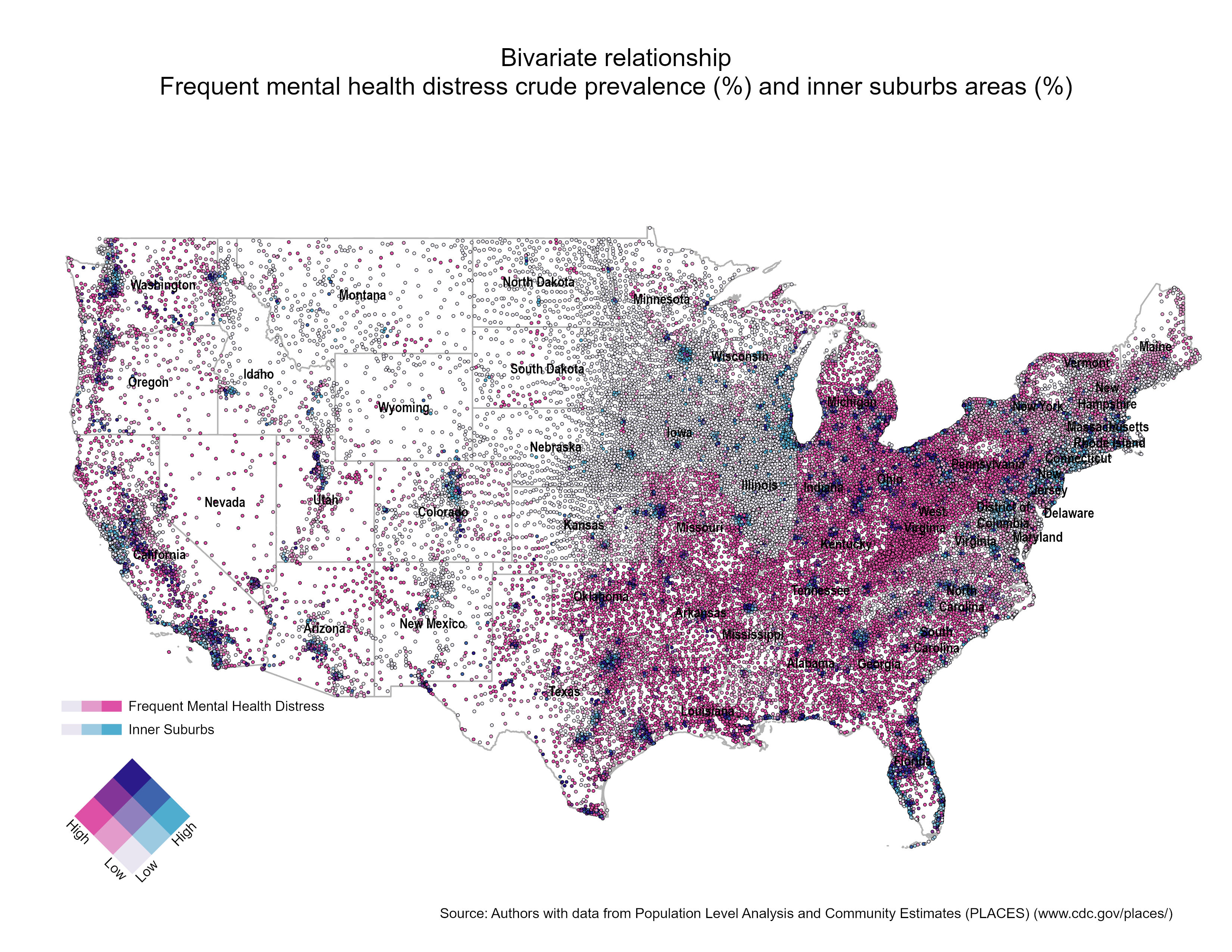

Supplement: Supplementary file 1 [file ijerph-20-05440-s001.zip › Supplementary Files and Appendix A/Figure 2G Inner Suburbs.jpg]

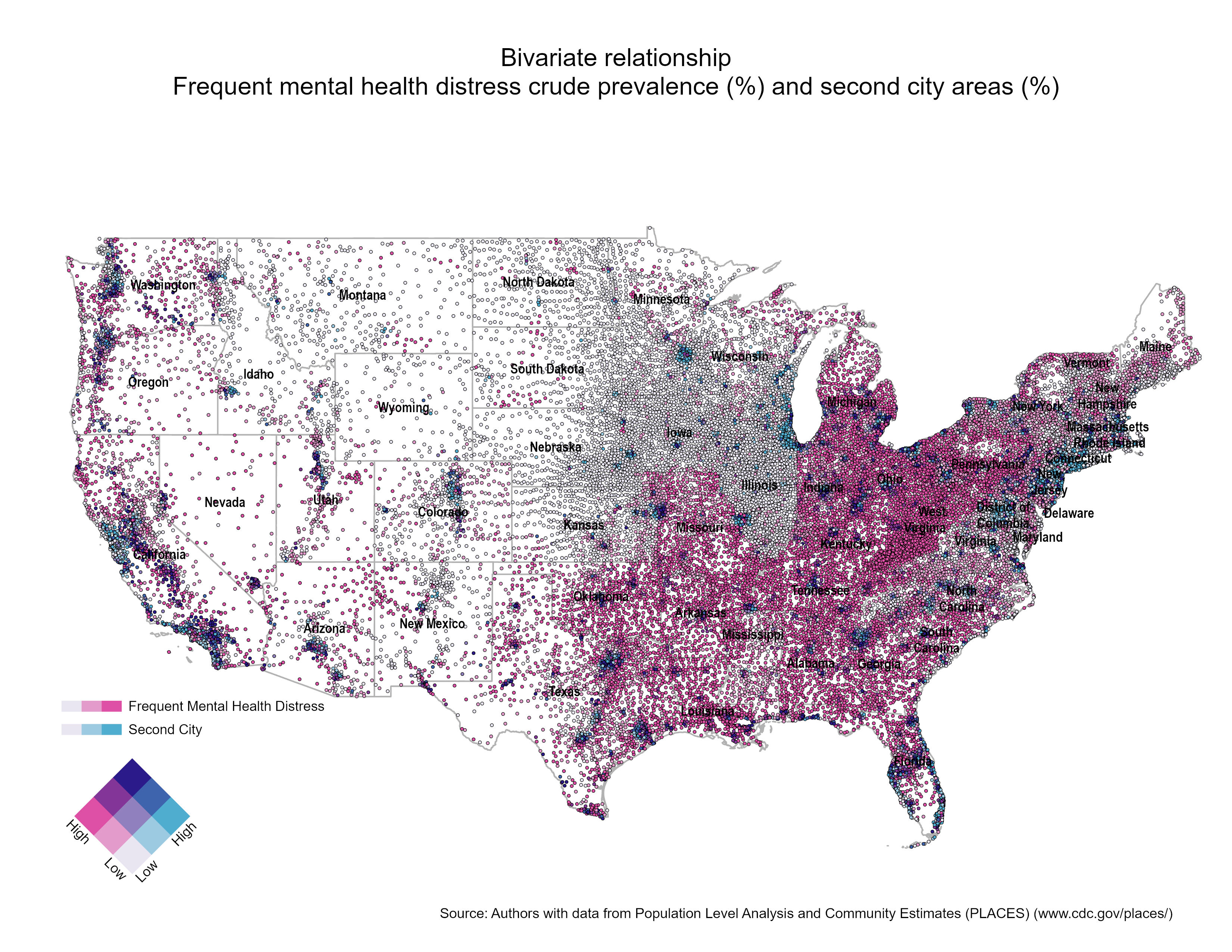

Supplement: Supplementary file 1 [file ijerph-20-05440-s001.zip › Supplementary Files and Appendix A/Figure 2H Second City.jpg]

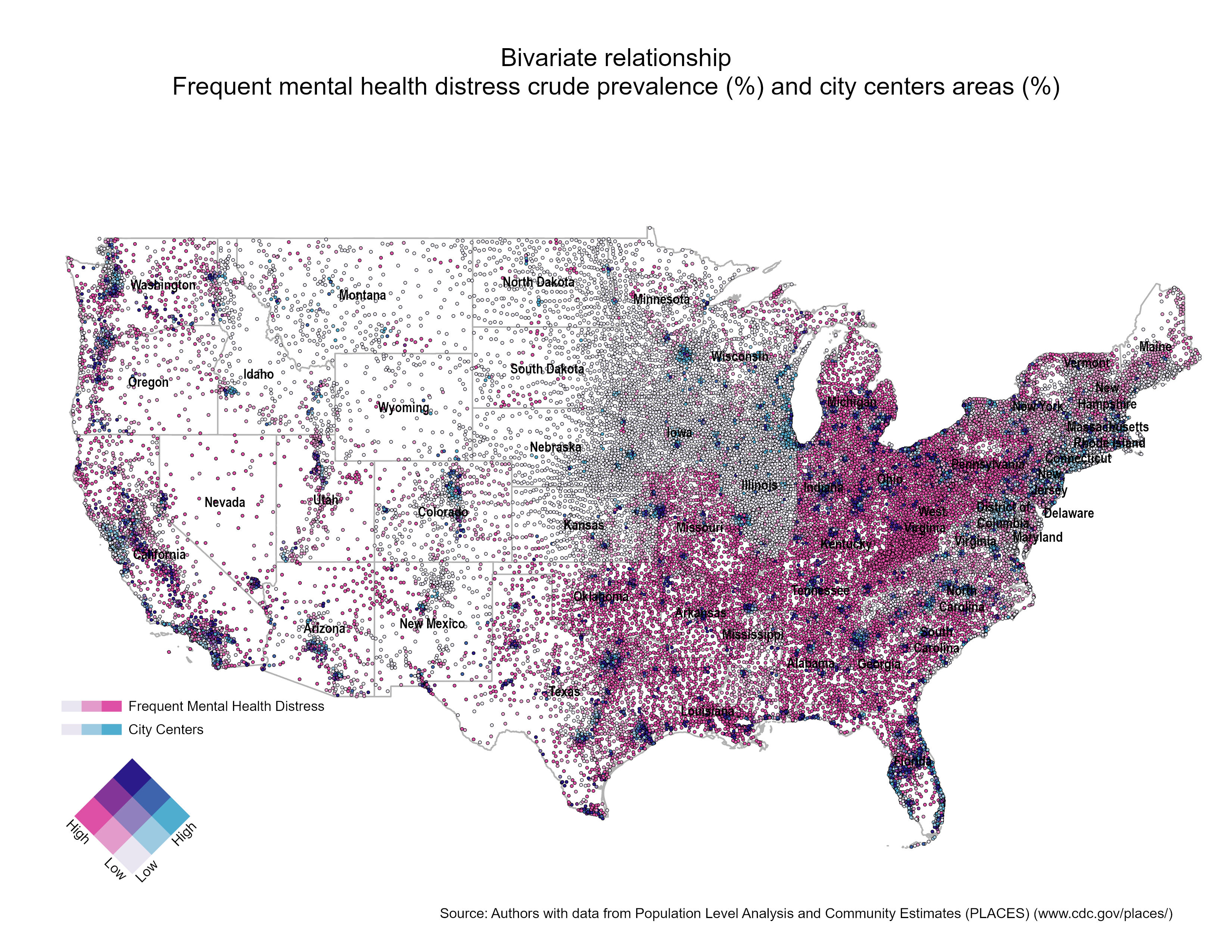

Supplement: Supplementary file 1 [file ijerph-20-05440-s001.zip › Supplementary Files and Appendix A/Figure 2I City Centers.jpg]

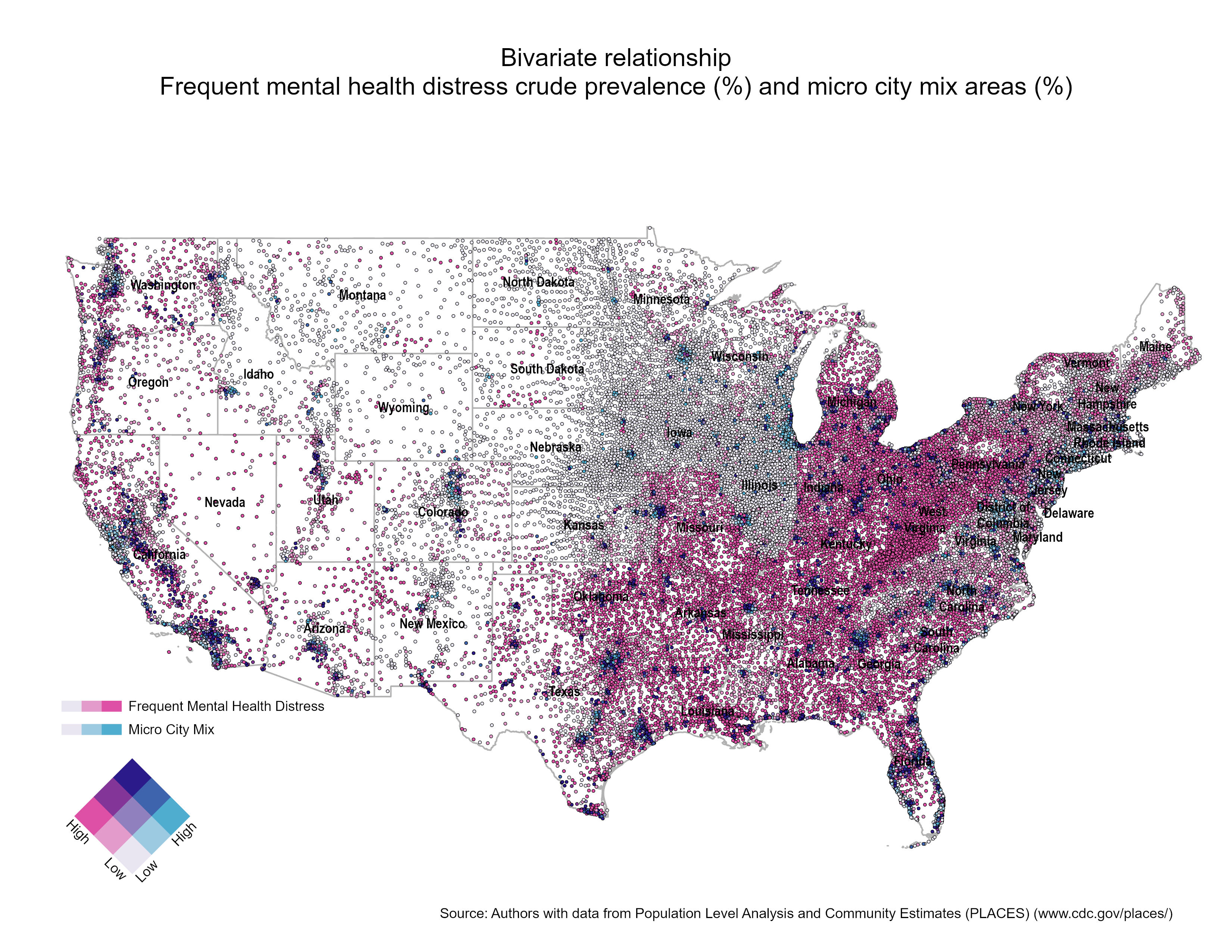

Supplement: Supplementary file 1 [file ijerph-20-05440-s001.zip › Supplementary Files and Appendix A/Figure 2J Micro City Mix.jpg]

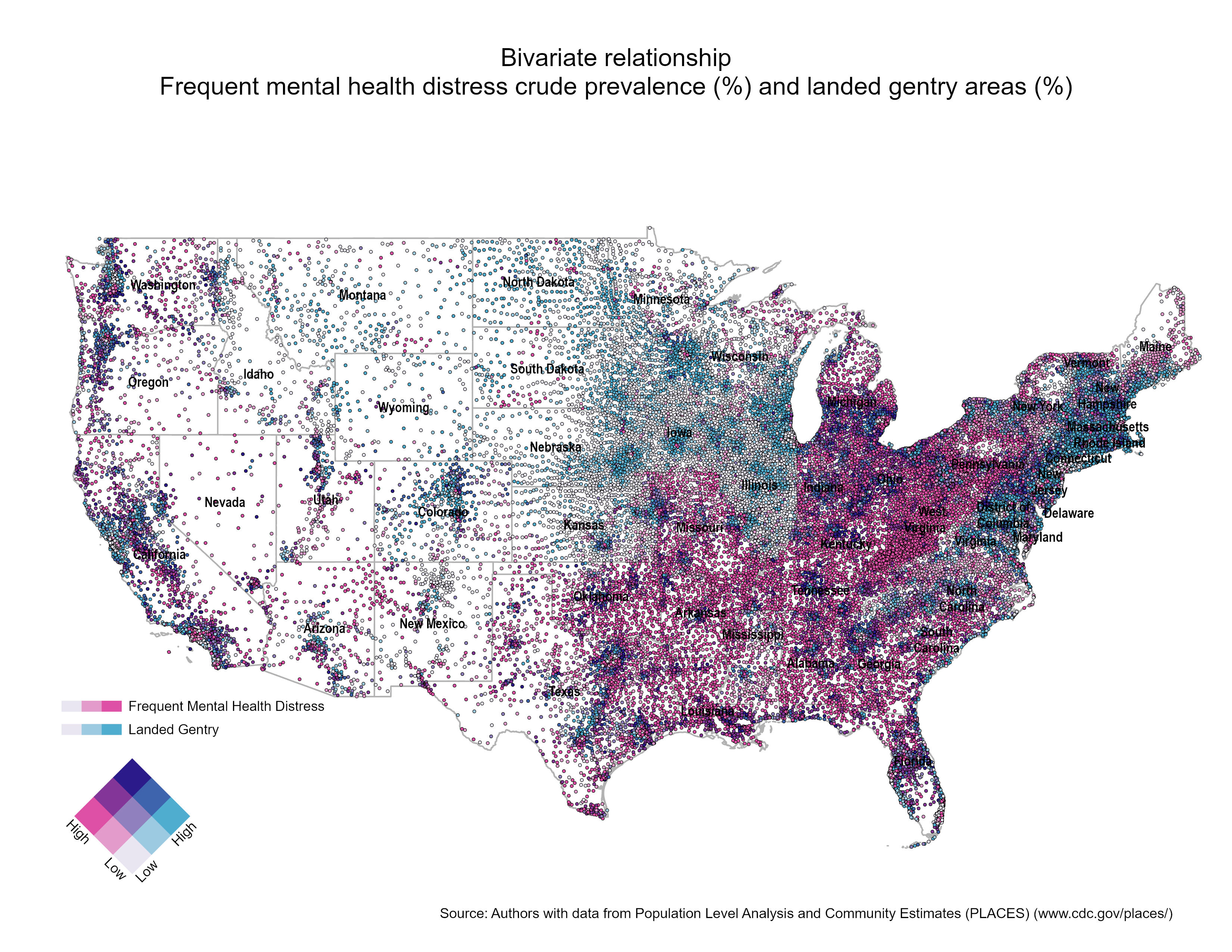

Supplement: Supplementary file 1 [file ijerph-20-05440-s001.zip › Supplementary Files and Appendix A/Figure 2K Landed Gentry.jpg]

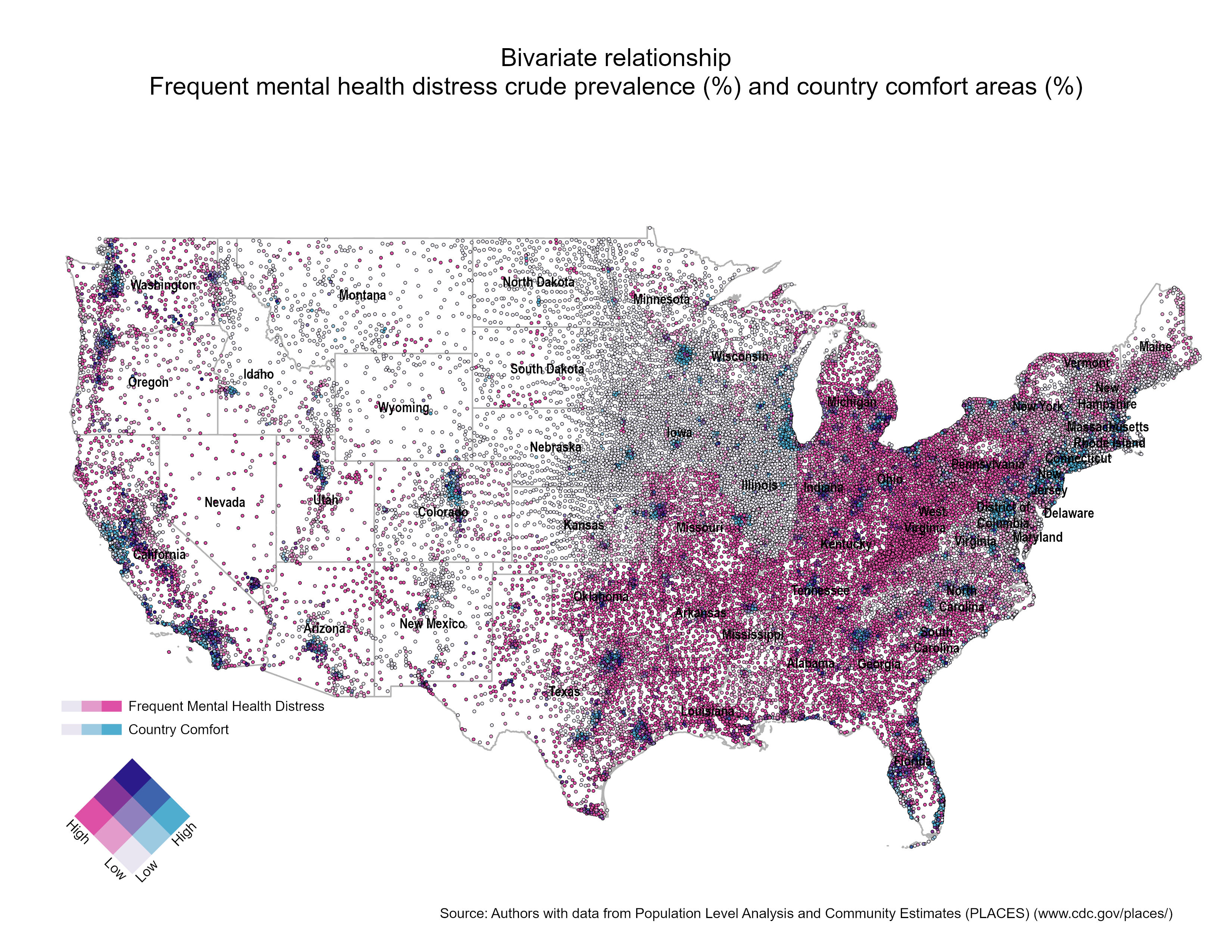

Supplement: Supplementary file 1 [file ijerph-20-05440-s001.zip › Supplementary Files and Appendix A/Figure 2L Country Comfort.jpg]

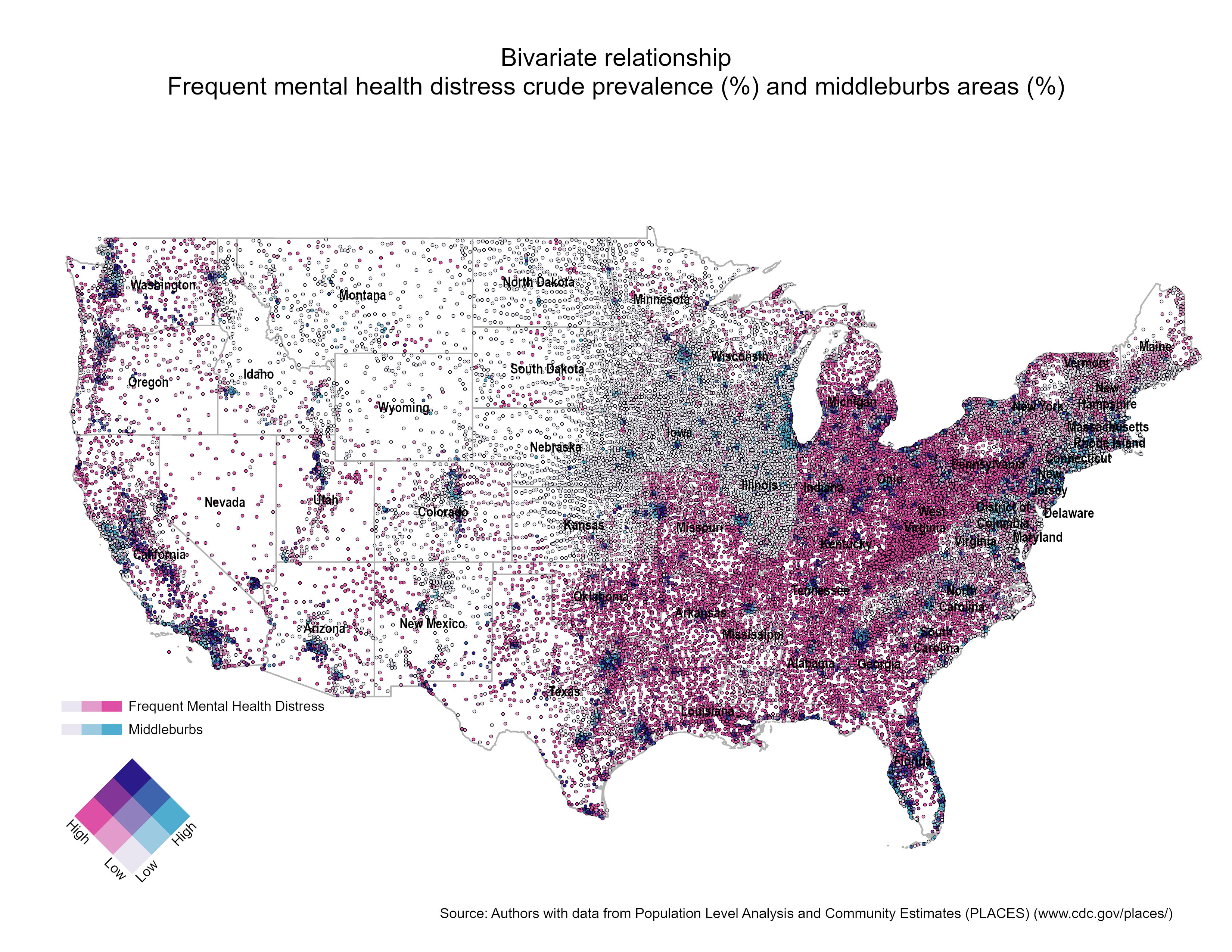

Supplement: Supplementary file 1 [file ijerph-20-05440-s001.zip › Supplementary Files and Appendix A/Figure 2M Middle America.jpg]

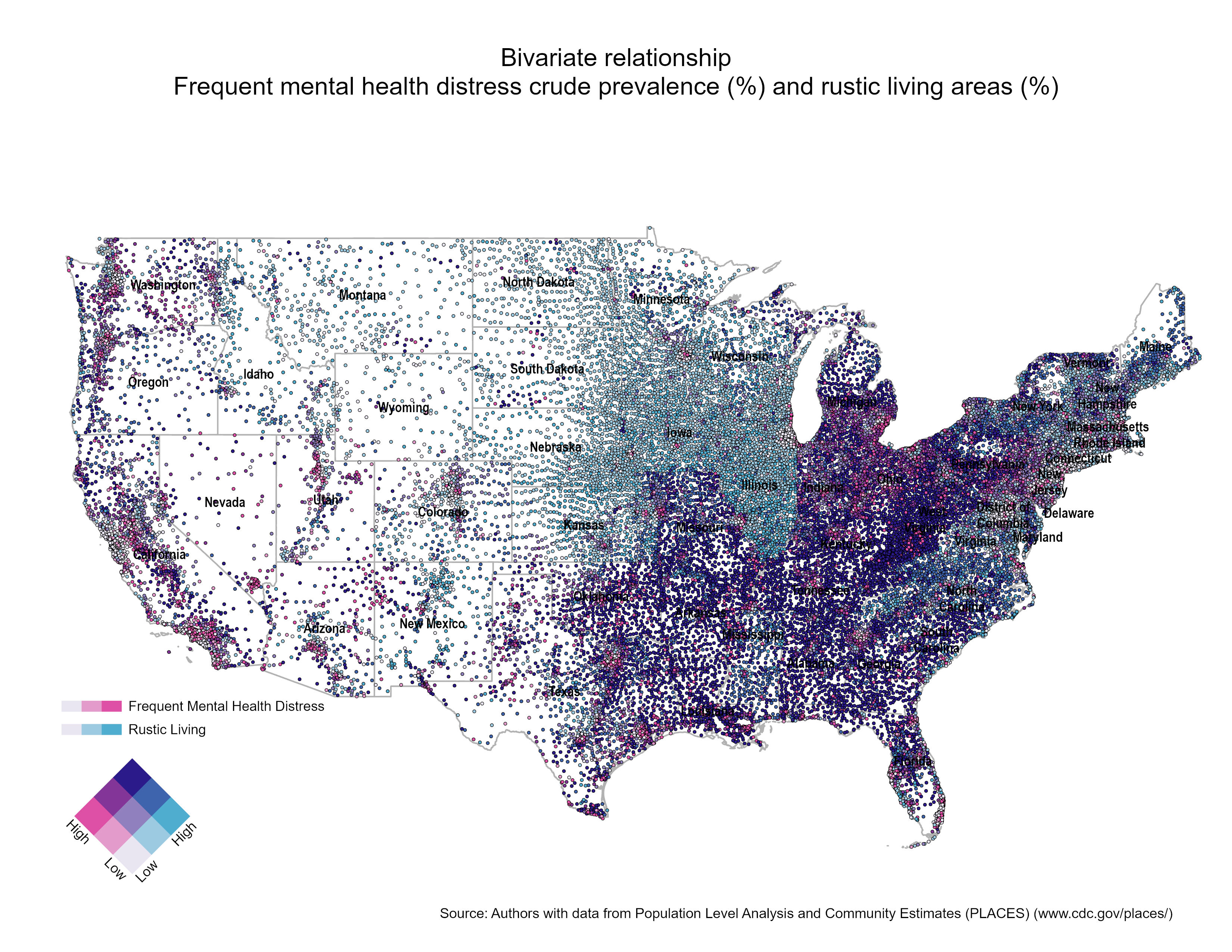

Supplement: Supplementary file 1 [file ijerph-20-05440-s001.zip › Supplementary Files and Appendix A/Figure 2N Rustic Living.jpg]

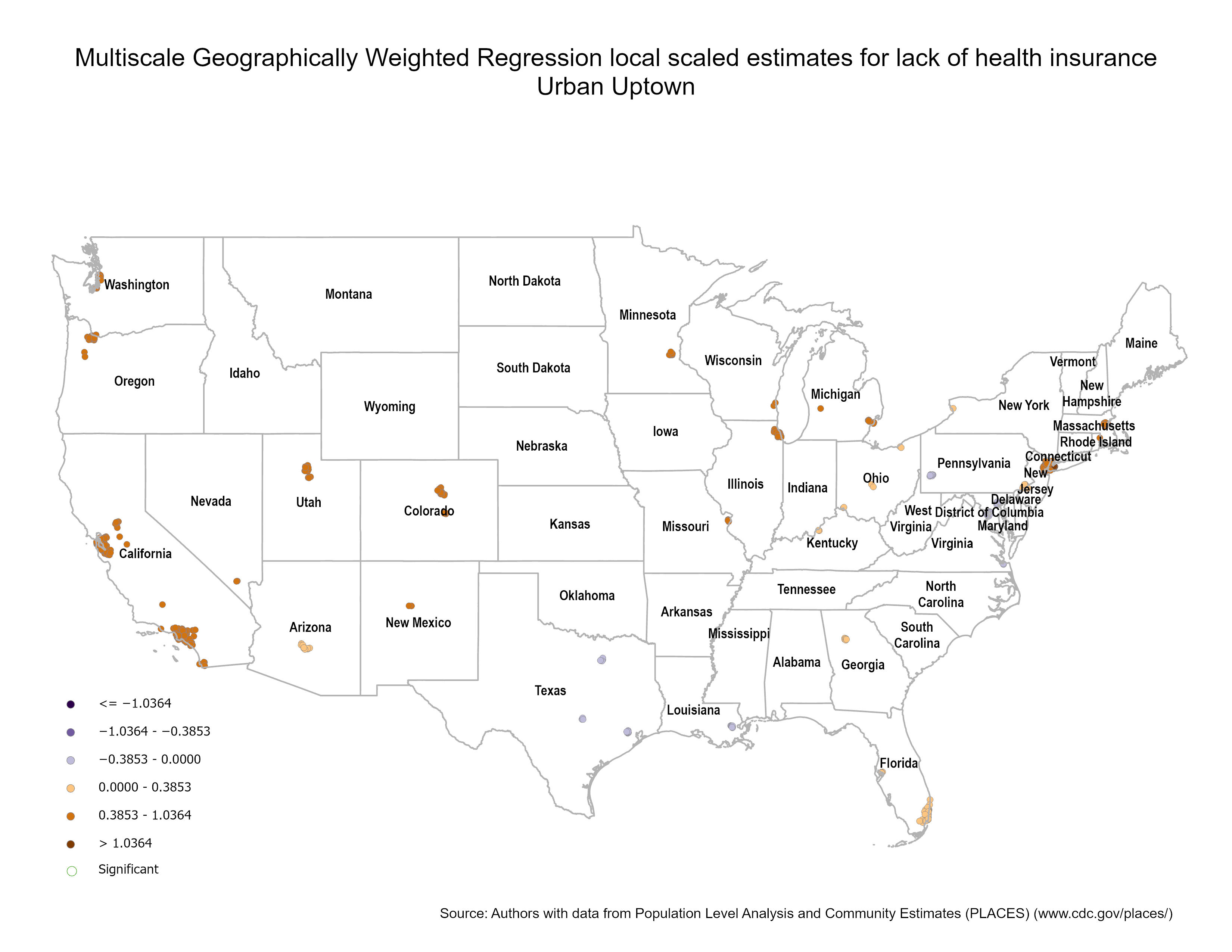

Supplement: Supplementary file 1 [file ijerph-20-05440-s001.zip › Supplementary Files and Appendix A/Figure 3A MGWRU1.jpg]

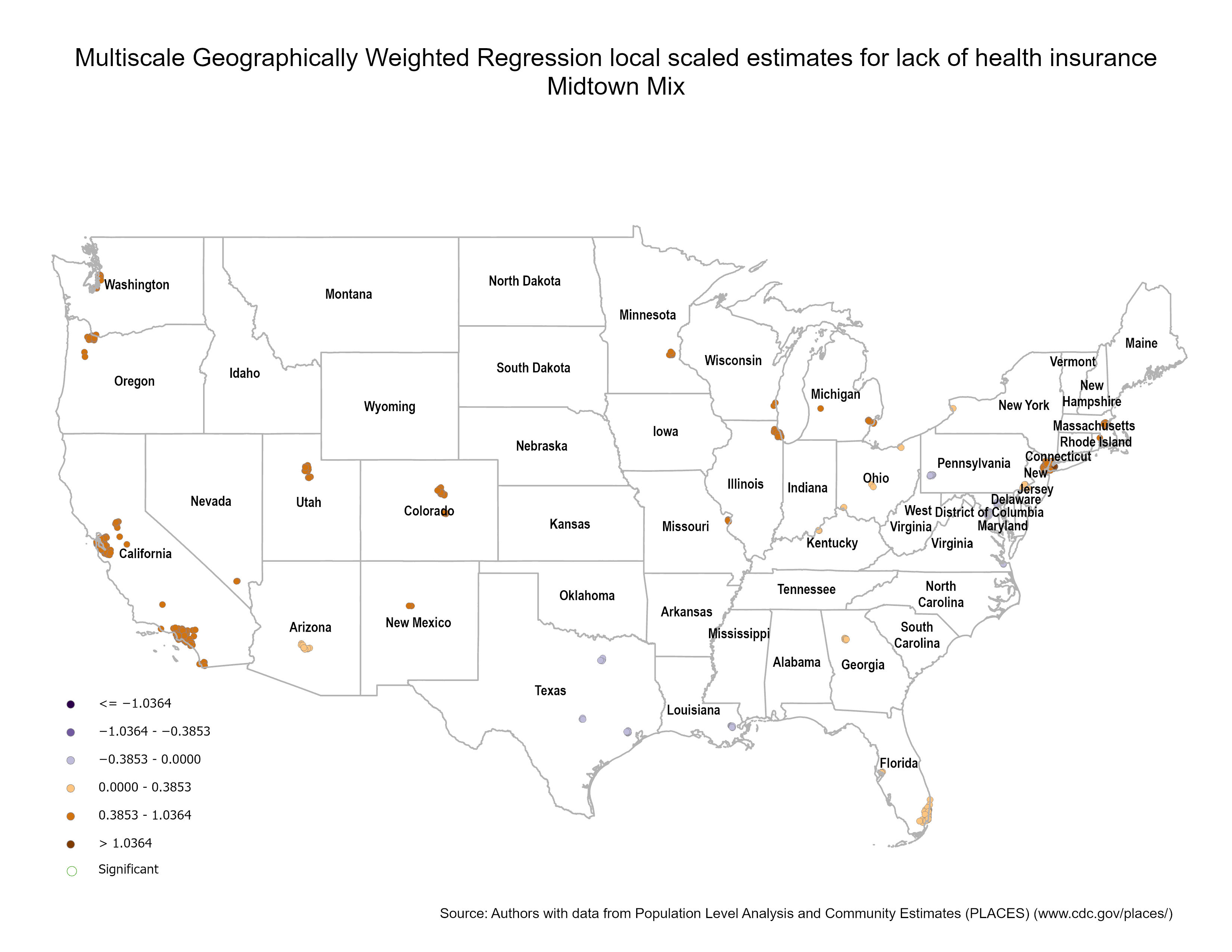

Supplement: Supplementary file 1 [file ijerph-20-05440-s001.zip › Supplementary Files and Appendix A/Figure 3B MGWRU2.jpg]

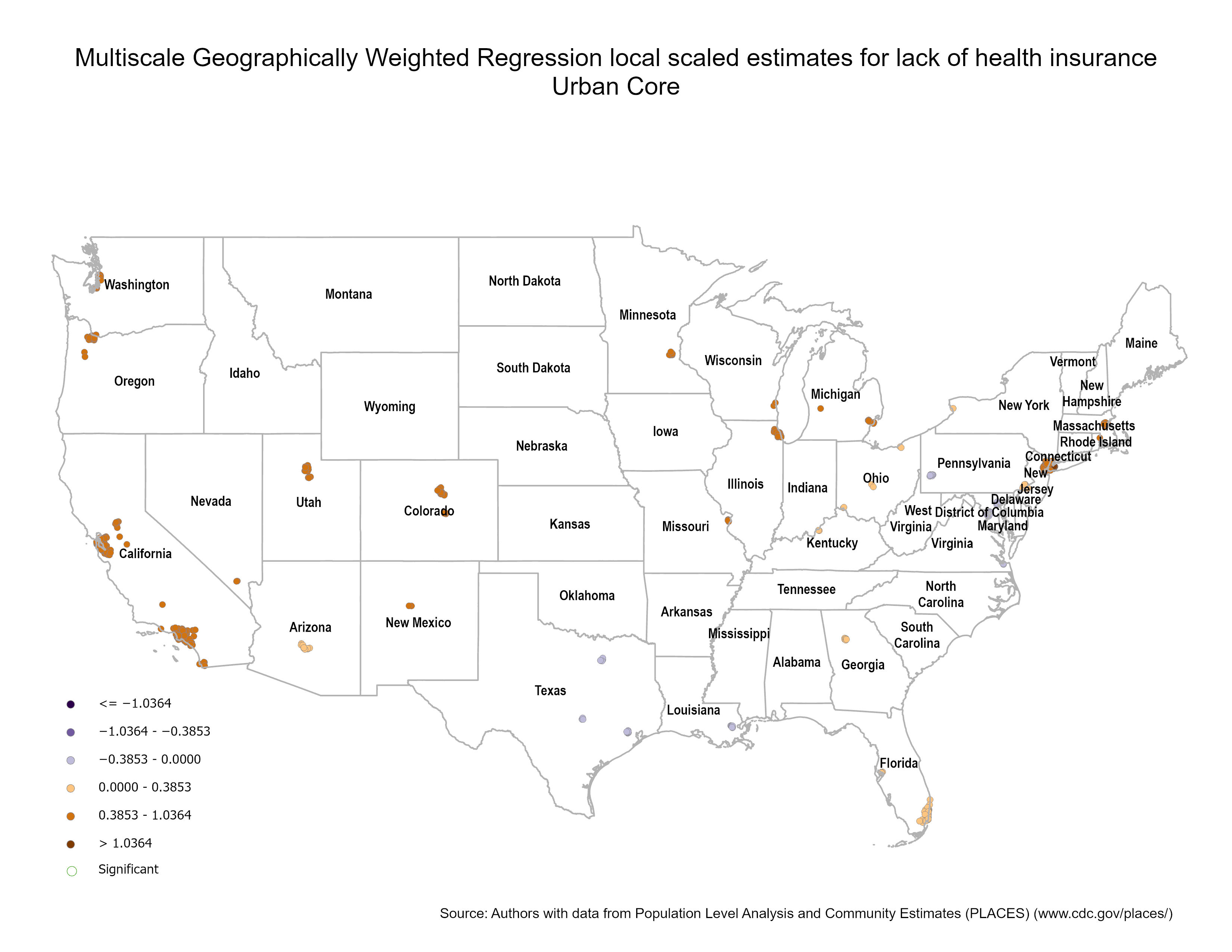

Supplement: Supplementary file 1 [file ijerph-20-05440-s001.zip › Supplementary Files and Appendix A/Figure 3C MGWRU3.jpg]

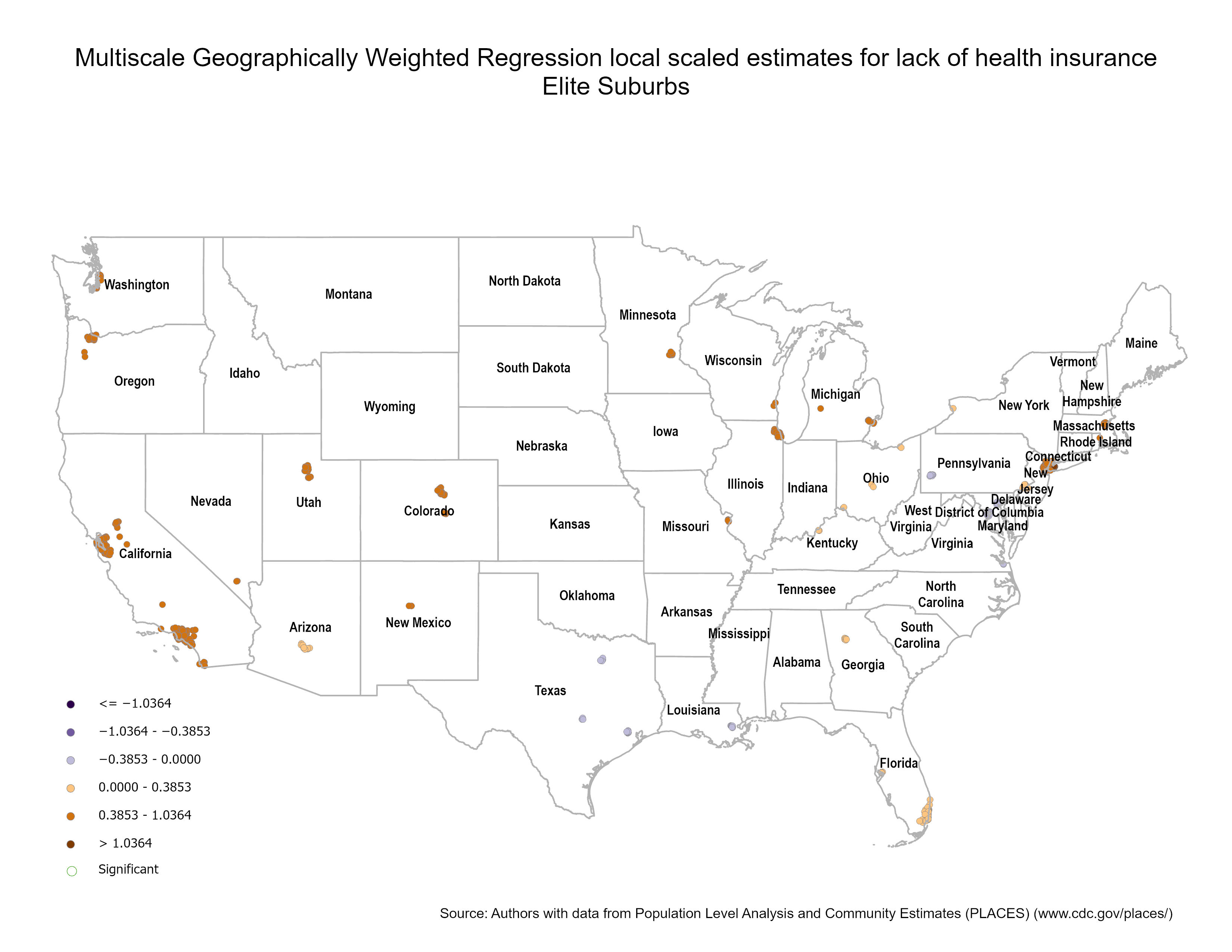

Supplement: Supplementary file 1 [file ijerph-20-05440-s001.zip › Supplementary Files and Appendix A/Figure 3D MGWRS1.jpg]

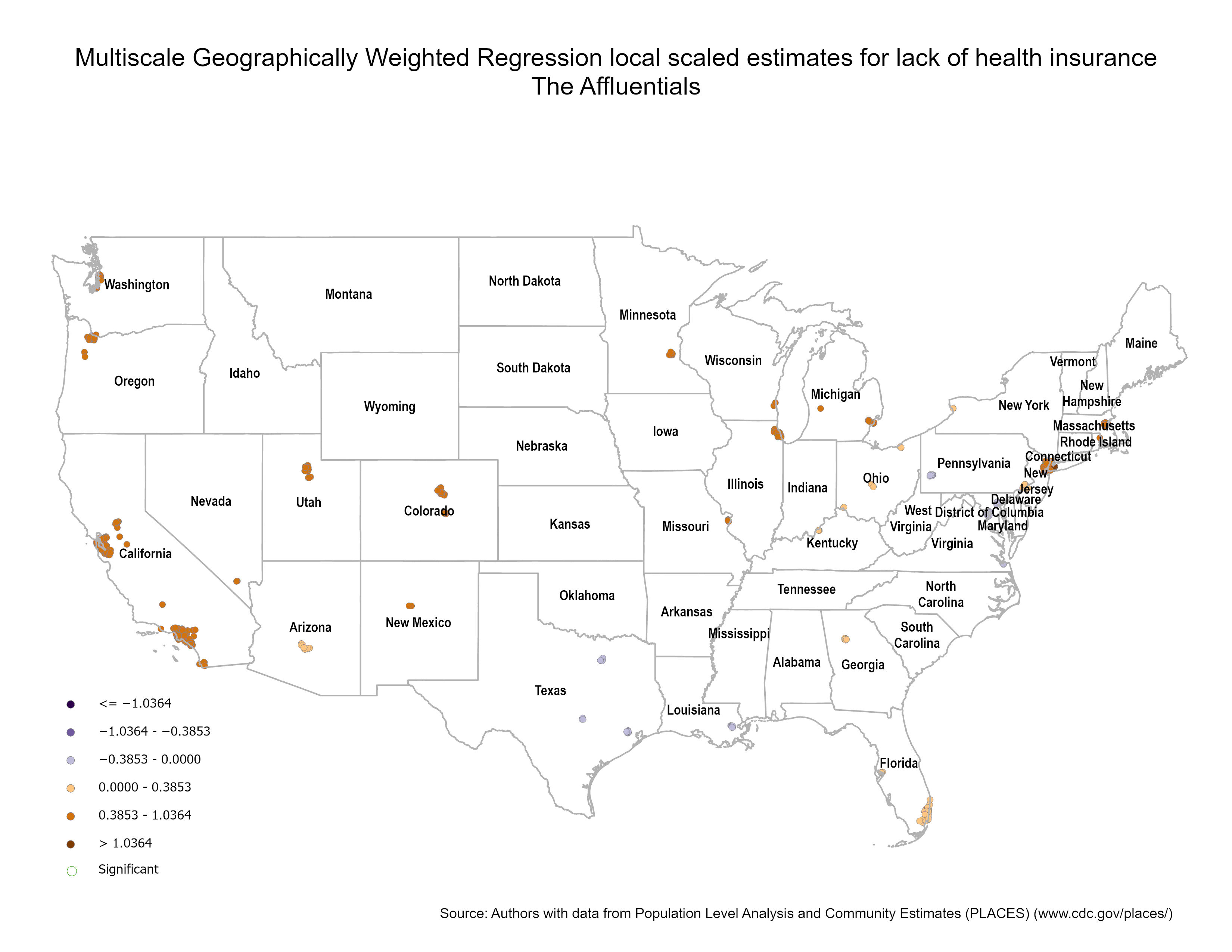

Supplement: Supplementary file 1 [file ijerph-20-05440-s001.zip › Supplementary Files and Appendix A/Figure 3E MGWRS2.jpg]

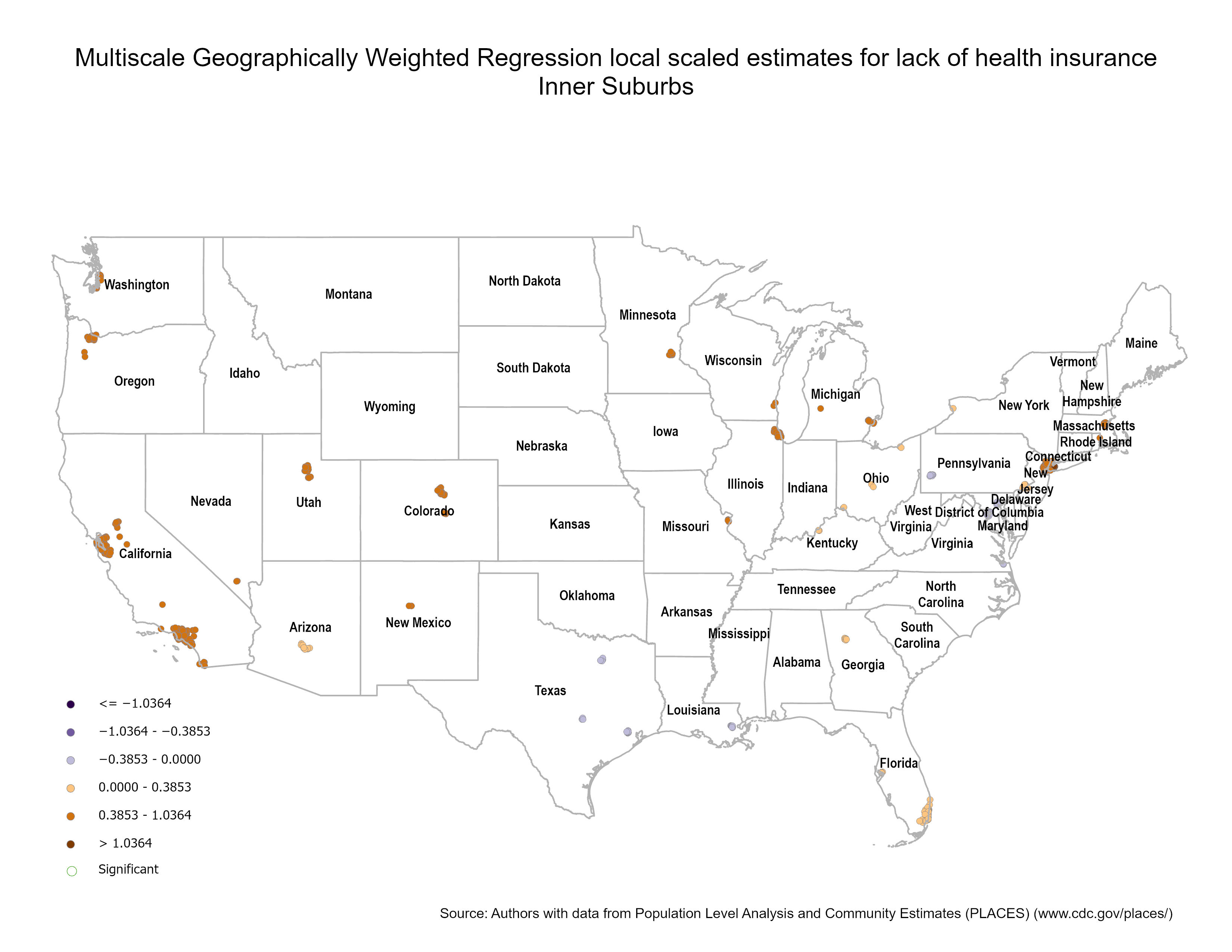

Supplement: Supplementary file 1 [file ijerph-20-05440-s001.zip › Supplementary Files and Appendix A/Figure 3G MGWRS4.jpg]

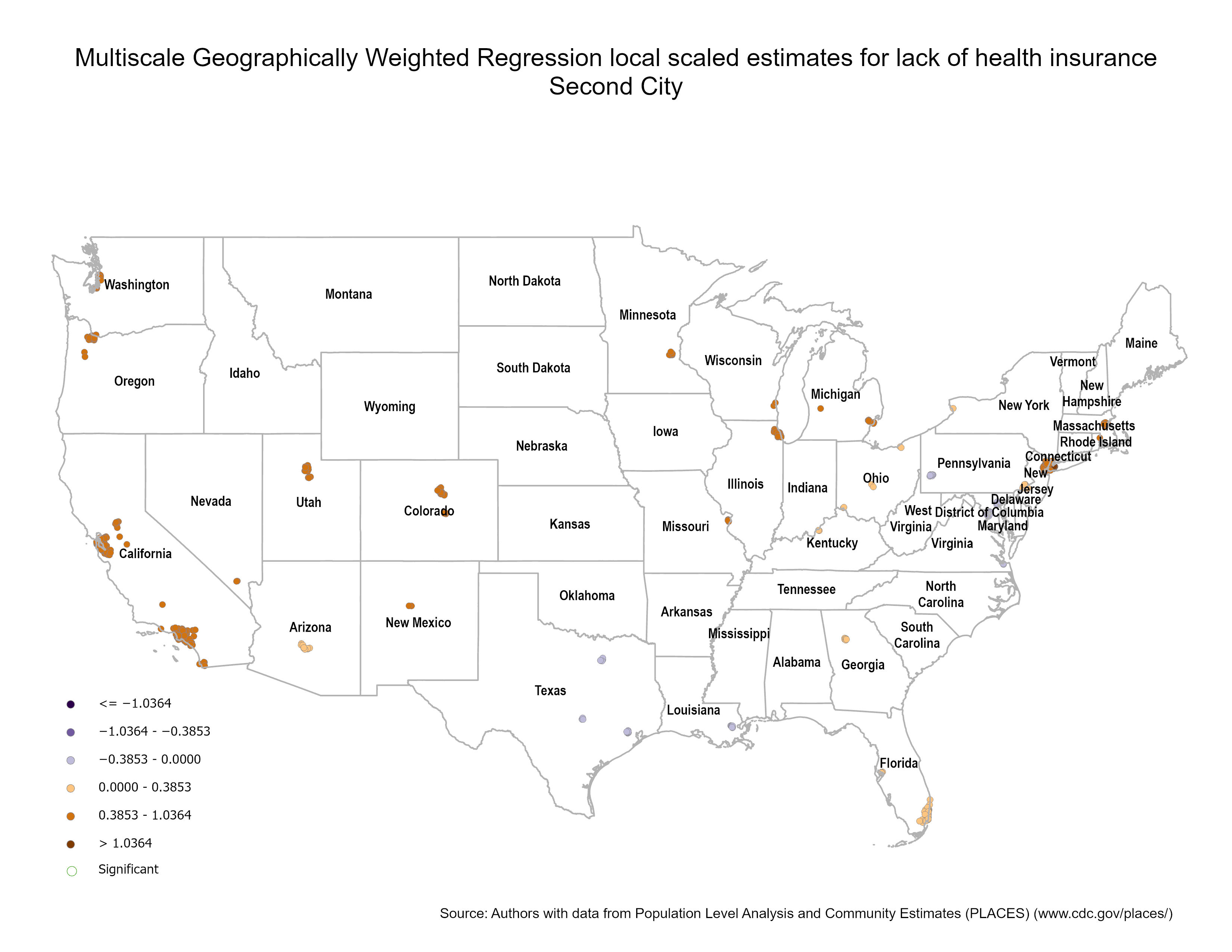

Supplement: Supplementary file 1 [file ijerph-20-05440-s001.zip › Supplementary Files and Appendix A/Figure 3H MGWRC1.jpg]

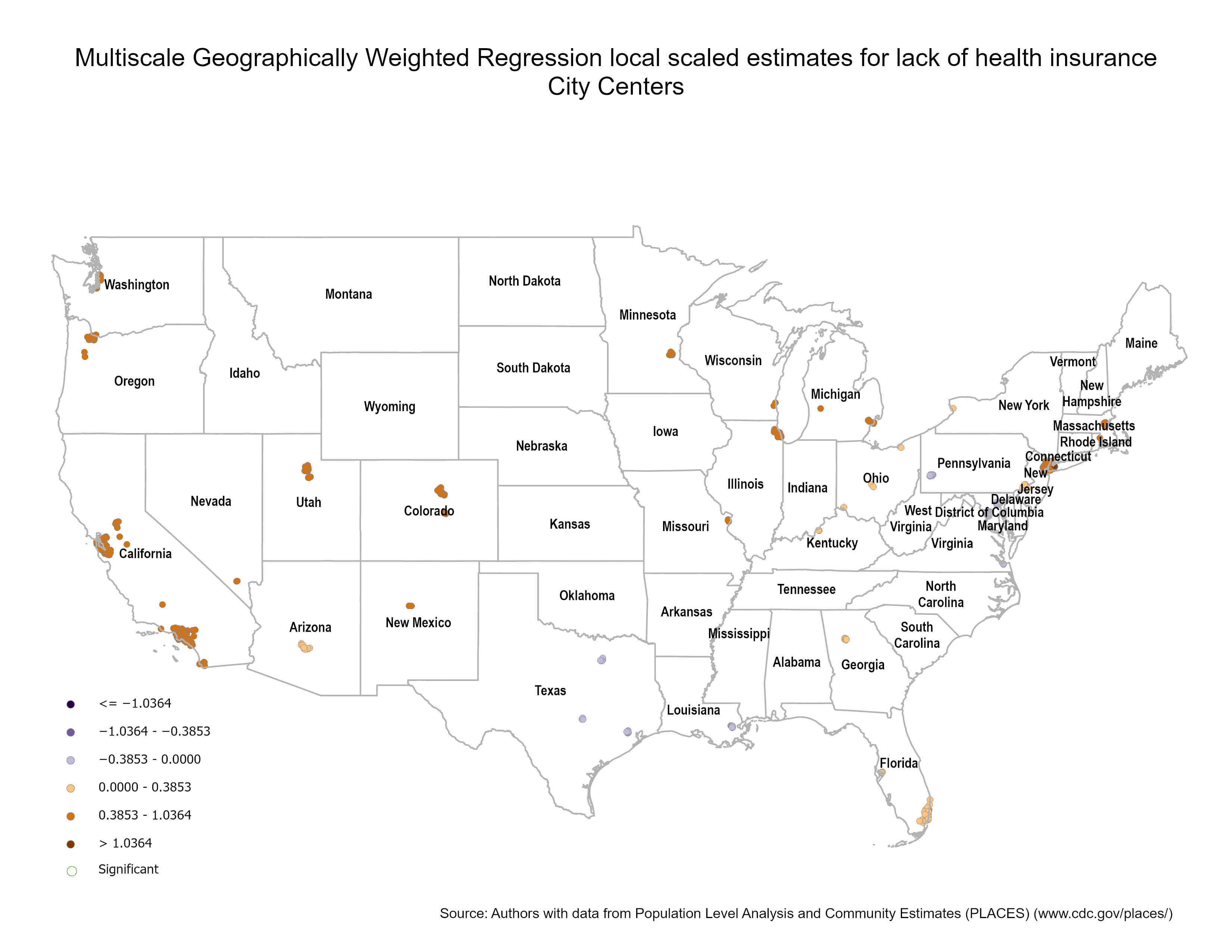

Supplement: Supplementary file 1 [file ijerph-20-05440-s001.zip › Supplementary Files and Appendix A/Figure 3I MGWRC2.jpg]

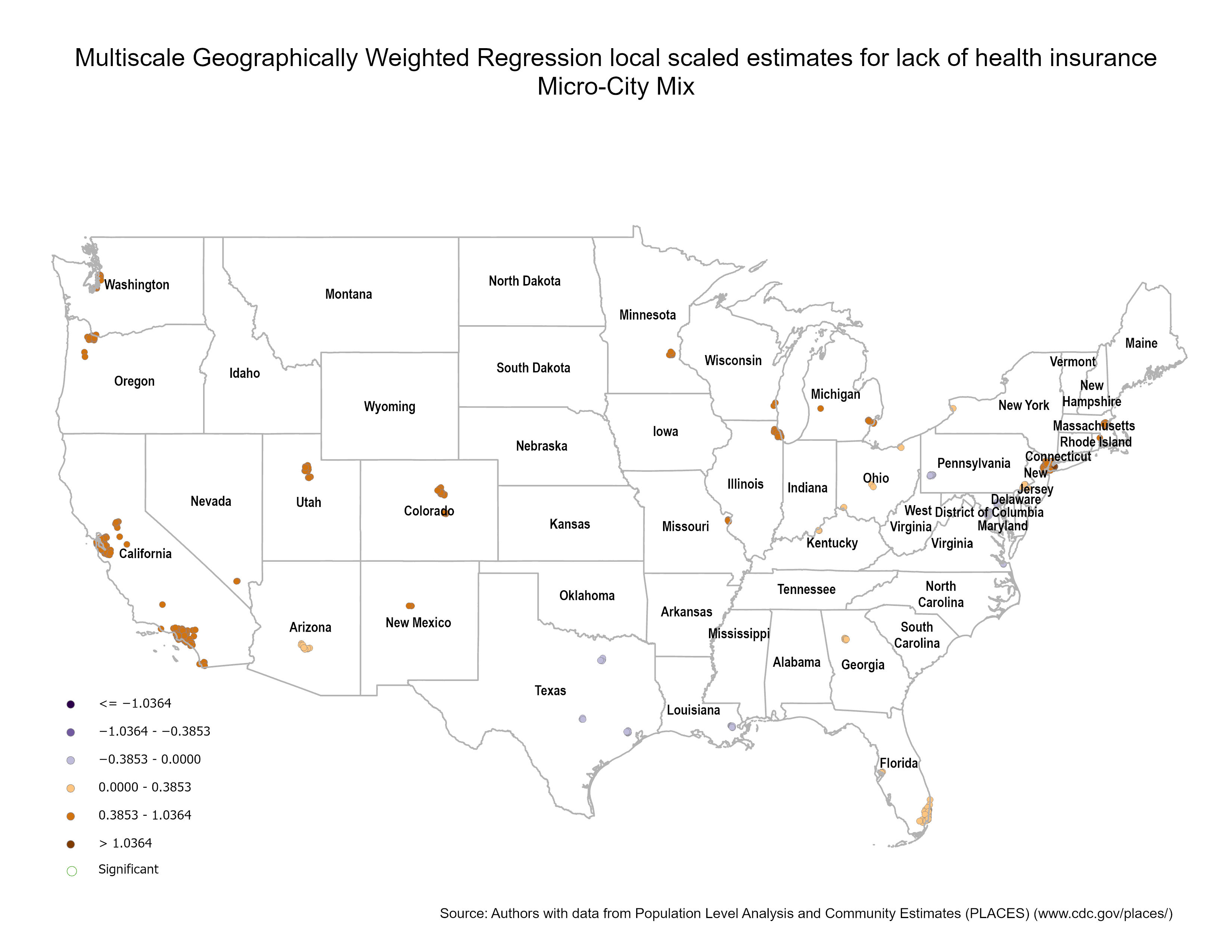

Supplement: Supplementary file 1 [file ijerph-20-05440-s001.zip › Supplementary Files and Appendix A/Figure 3J MGWRC3.jpg]

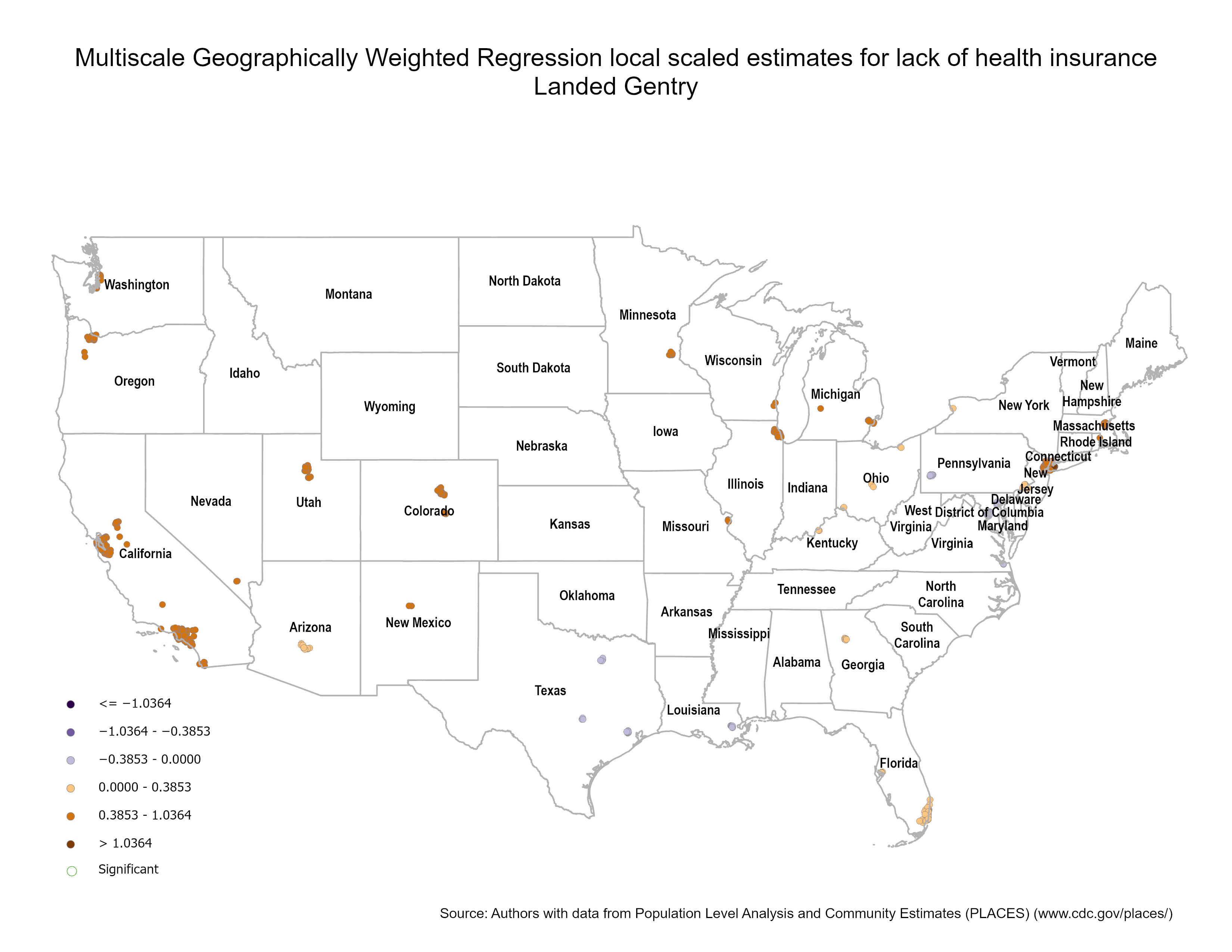

Supplement: Supplementary file 1 [file ijerph-20-05440-s001.zip › Supplementary Files and Appendix A/Figure 3K MGWRT1.jpg]

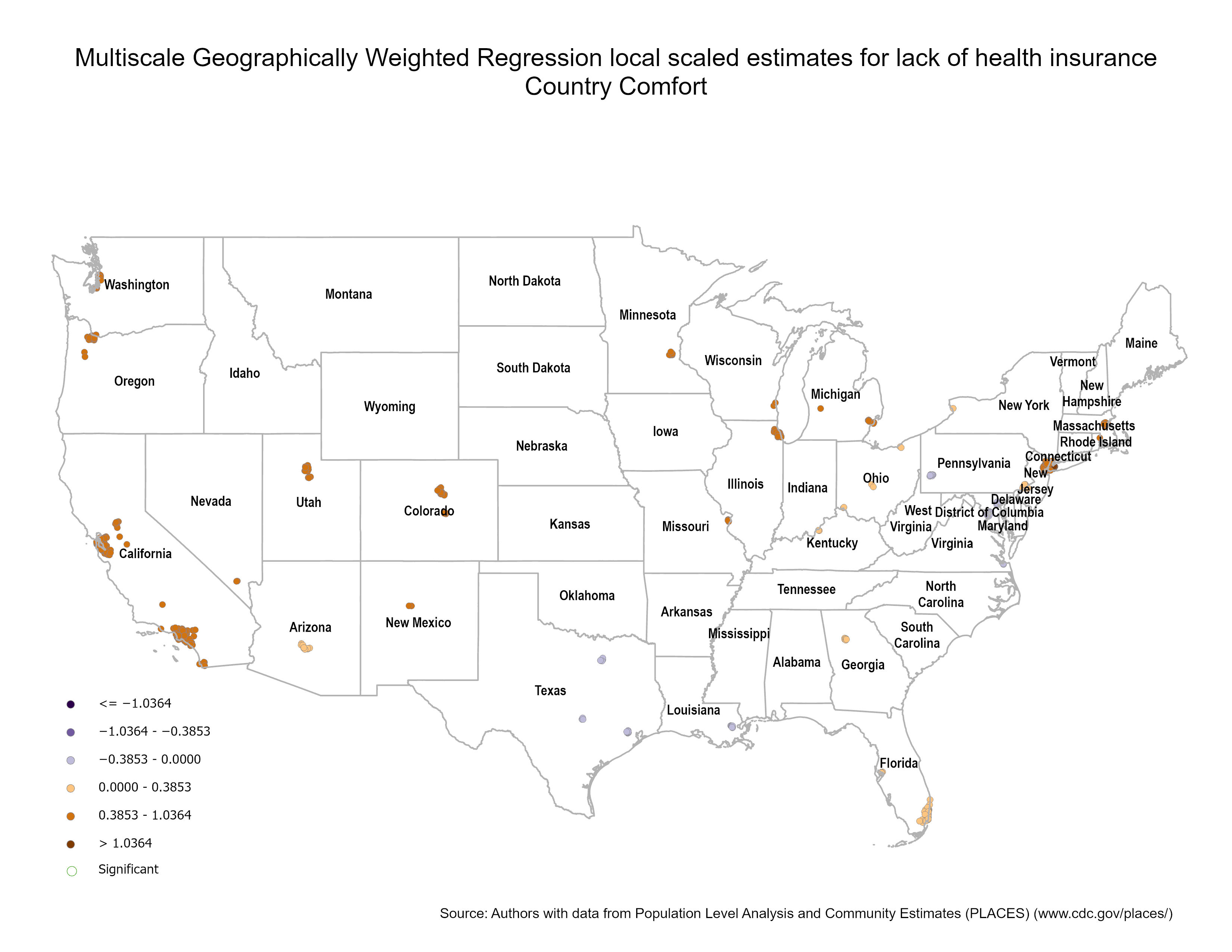

Supplement: Supplementary file 1 [file ijerph-20-05440-s001.zip › Supplementary Files and Appendix A/Figure 3L MGWRT2.jpg]

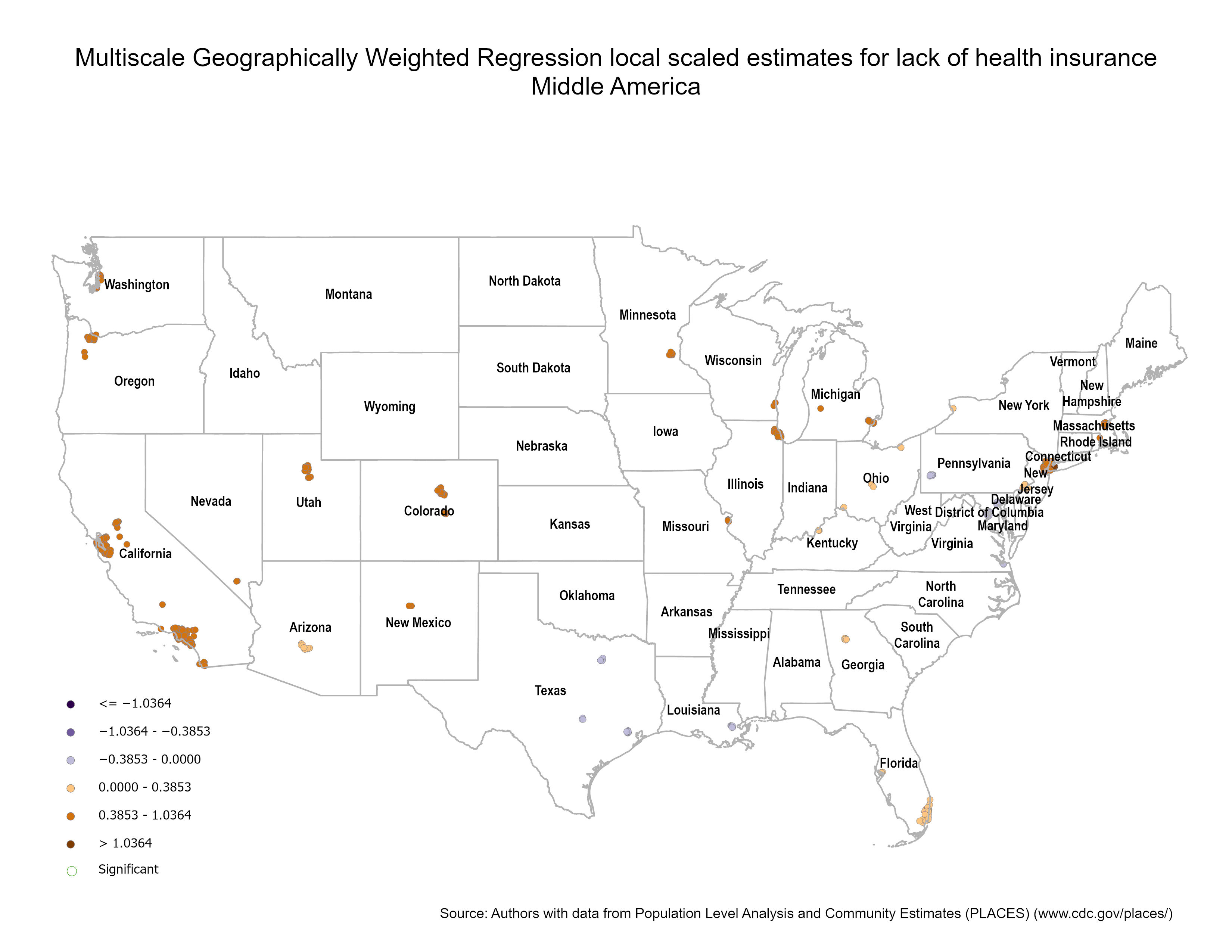

Supplement: Supplementary file 1 [file ijerph-20-05440-s001.zip › Supplementary Files and Appendix A/Figure 3M MGWRT3.jpg]

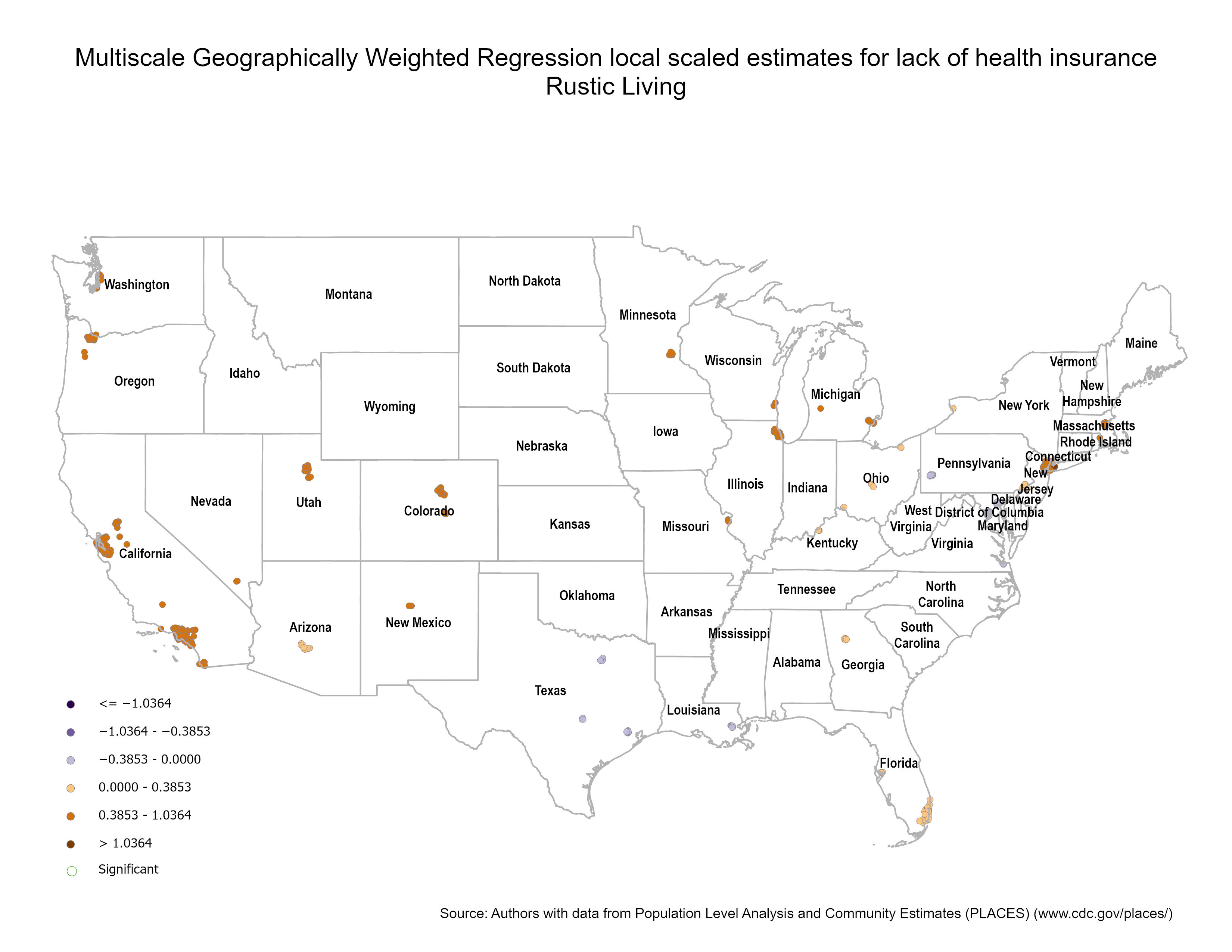

Supplement: Supplementary file 1 [file ijerph-20-05440-s001.zip › Supplementary Files and Appendix A/Figure 3N MGWRT4.jpg]

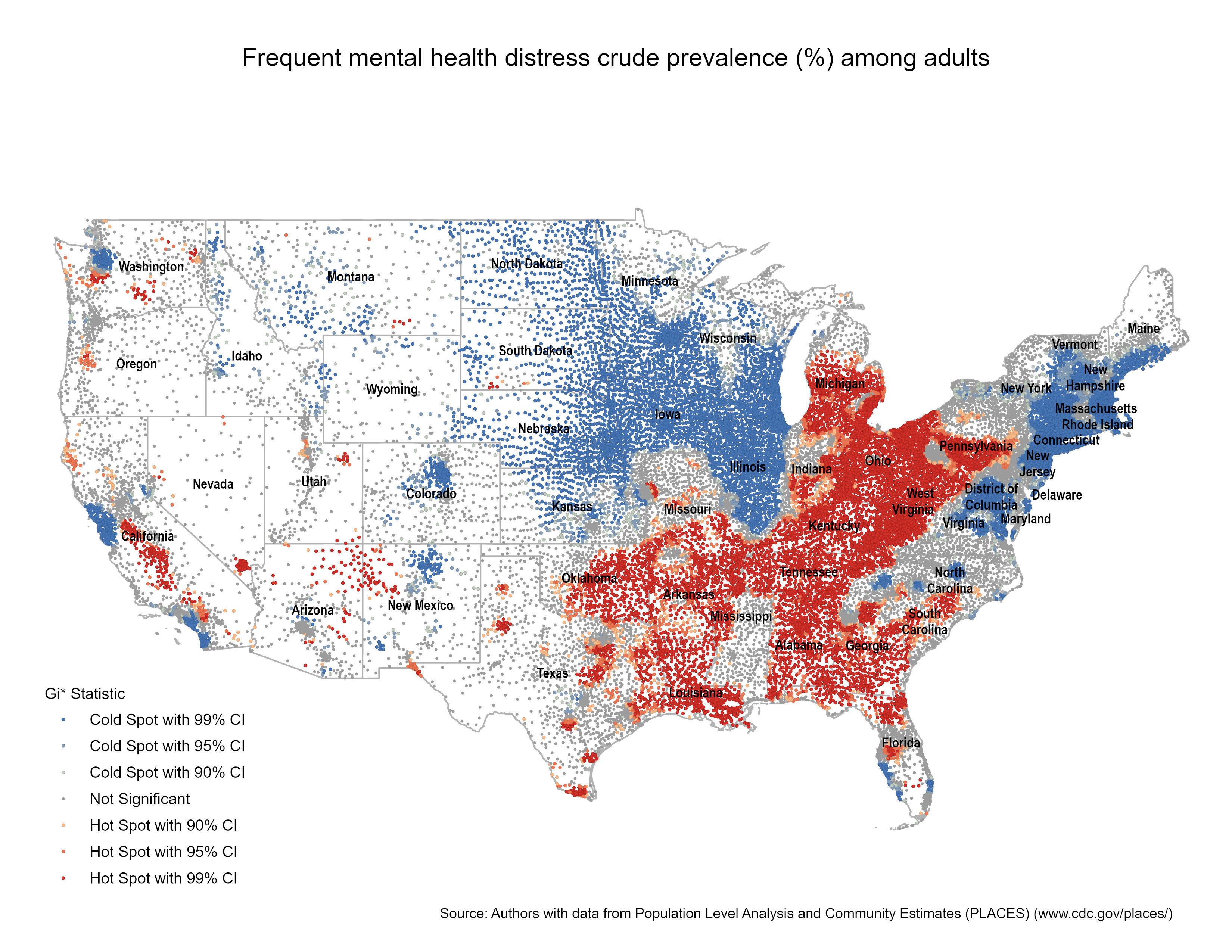

Supplement: Supplementary file 1 [file ijerph-20-05440-s001.zip › Supplementary Files and Appendix A/Figure S1.jpg]
